# CAG-encoded polyglutamine length polymorphism in the human genome

**DOI:** 10.1186/1471-2164-8-126

**Published:** 2007-05-22

**Authors:** Stefanie L Butland, Rebecca S Devon, Yong Huang, Carri-Lyn Mead, Alison M Meynert, Scott J Neal, Soo Sen Lee, Anna Wilkinson, George S Yang, Macaire MS Yuen, Michael R Hayden, Robert A Holt, Blair R Leavitt, BF Francis Ouellette

**Affiliations:** 1 UBC Bioinformatics Centre, Michael Smith Laboratories, University of British Columbia, Vancouver, Canada; 2 Centre for Molecular Medicine and Therapeutics, Child and Family Research Institute, Department of Medical Genetics, University of British Columbia, Vancouver, Canada; 3 Canada's Michael Smith Genome Sciences Centre, British Columbia Cancer Agency, Vancouver, Canada; 4 Department of Medical Genetics, University of British Columbia, Vancouver, Canada; 5 Department of Psychiatry, University of British Columbia, Vancouver, Canada

## Abstract

**Background:**

Expansion of polyglutamine-encoding CAG trinucleotide repeats has been identified as the pathogenic mutation in nine different genes associated with neurodegenerative disorders. The majority of individuals clinically diagnosed with spinocerebellar ataxia do not have mutations within known disease genes, and it is likely that additional ataxias or Huntington disease-like disorders will be found to be caused by this common mutational mechanism. We set out to determine the length distributions of CAG-polyglutamine tracts for the entire human genome in a set of healthy individuals in order to characterize the nature of polyglutamine repeat length variation across the human genome, to establish the background against which pathogenic repeat expansions can be detected, and to prioritize candidate genes for repeat expansion disorders.

**Results:**

We found that repeats, including those in known disease genes, have unique distributions of glutamine tract lengths, as measured by fragment analysis of PCR-amplified repeat regions. This emphasizes the need to characterize each distribution and avoid making generalizations between loci. The best predictors of known disease genes were occurrence of a long CAG-tract uninterrupted by CAA codons in their reference genome sequence, and high glutamine tract length variance in the normal population. We used these parameters to identify eight priority candidate genes for polyglutamine expansion disorders. Twelve CAG-polyglutamine repeats were invariant and these can likely be excluded as candidates. We outline some confusion in the literature about this type of data, difficulties in comparing such data between publications, and its application to studies of disease prevalence in different populations. Analysis of Gene Ontology-based functions of CAG-polyglutamine-containing genes provided a visual framework for interpretation of these genes' functions. All nine known disease genes were involved in DNA-dependent regulation of transcription or in neurogenesis, as were all of the well-characterized priority candidate genes.

**Conclusion:**

This publication makes freely available the normal distributions of CAG-polyglutamine repeats in the human genome. Using these background distributions, against which pathogenic expansions can be identified, we have begun screening for mutations in individuals clinically diagnosed with novel forms of spinocerebellar ataxia or Huntington disease-like disorders who do not have identified mutations within the known disease-associated genes.

## Background

Nine different neurodegenerative disorders are known to be caused by expansions of polyglutamine-encoding CAG trinucleotide (CAGpolyQ) repeats in the following genes: the *HD *gene in Huntington disease [1], *ATN1 *in dentatorubral pallidoluysian atrophy or Haw River syndrome [2,3], *AR *in spinal and bulbar muscular atrophy [4], *CACNA1A *in spinocerebellar ataxia SCA6 [5], *TBP *in SCA17 [6] and *ATXN1*, *2*, *3*, and *7 *in SCA1 [7], SCA2 [8-10], SCA3 (Machado-Joseph disease) [11], and SCA7 [12]. These disorders share similar clinical features which include selective neuronal degradation associated with a progressive neurological phenotype, but their respective causative genes appear to have little functional or structural similarity, suggesting that functional genomics approaches to identifying new gene-disease associations will not be useful. The repeat expansion mechanism of pathogenesis is a shared molecular feature, and this form of mutation has only been exhaustively ruled out for a few familial forms of SCA, and has not been examined at all for the majority of patients who present with SCA or HD-like disorders.

Despite recent advances in molecular diagnosis, the majority of individuals clinically diagnosed with SCA do not have identified mutations within the known disease-associated genes [13]. There are 28 genetically distinct SCAs identified by the Human Gene Nomenclature Committee (HGNC) [14], but only 13 causative genes are known. Six genes cause SCA by CAGpolyQ expansions, but the remaining 15 clinically-defined forms of SCA have no known genetic mutation associated with them, and the search for causative genes continues. It is likely that some of these forms of SCA will be found to be caused by this common mutational mechanism. Candidate genes for SCA and HD-like disorders can be identified using a whole-genome screening approach based on the computational identification of a common sequence we have termed a Genomic Mutational Signature (GeMS). GeMS are sequence patterns occurring in the normal genome that, when mutated, cause disease – in this case CAG trinucleotide repeats that encode an extended tract of glutamine residues in the protein. A significant advantage of this approach is that novel candidate disease genes are identified and can then be screened for mutations in single cases. This approach is not constrained by any requirement for additional family members, additional affected patients, nor is a detailed family history required.

Partial lists of CAGpolyQ-containing genes identified using classical [15-20] or computational methods [21-24] have been published. Screening for CAG expansions in one such gene list, in patients with hereditary ataxias, led directly to the discovery of the causative gene for dentatorubral pallidoluysian atrophy [2,16]. To date, there has been no complete genome-wide analysis of the distributions of CAGpolyQ repeat lengths in a control population in order to set the baseline from which to detect expansions. Studies on a limited number of genes have revealed that different genes have very different polyglutamine tract (Q-tract) length distributions with some invariant (*CREBBP*) [25] some bimodal (*ATXN3*) [26], some very narrow (*ATXN2*) [26] and some broad distributions (*AR*, *ATN1*, *SMARCA2 *and *THAP11*) [26-28].

### The molecular nature of polyglutamine repeats

The amino acid glutamine (Q) is encoded by CAG and CAA trinucleotides. Q-tracts in proteins are typically encoded by mixtures of these two codons while expanded Q-tracts in disease-causing genes are typically composed of long uninterrupted repeats of the CAG trinucleotide only. Long uninterrupted CAG repeats are known to be a substrate for expansion mutation by a variety of mechanisms. The underlying process is currently thought to involve the generation of abnormal DNA structures induced by factors such as replication slippage, DNA repair and recombination, that can contribute to repeat instability acting either separately or in combination [29-32] and these mutations underlie pathogenic expansions [33] and genetic anticipation [34,35]. Q-tracts encoded by mixtures of CAG and CAA codons, however, are less prone to suffer expansions [30,36,37]. The precise nucleotide sequence of a repeat tract determines a particular allele's susceptibility to large expansion mutations, while the amino acid sequence – the Q-tract – in the context of the whole protein determines the effect of a length change on molecular and clinical phenotypes.

### Characteristics of known disease genes

One motivation for this research was to enable us to prioritize candidate genes for polyglutamine expansion disorders. Thus, we sought to identify hallmarks among the known disease genes to which we could compare our data on CAGpolyQ genes not yet associated with disease. Disease-causing CAGpolyQ-containing genes tend to be considered a homogeneous group in terms of their repeats, with an often-cited pathogenic threshold of about 35 glutamines. In fact, a closer look at normal and pathogenic characteristics of each reveals their unique qualities. *ATXN2 *has a remarkably narrow distribution of Q-tract lengths with very few alleles longer or shorter than the modal length of 22Q [26,37]. In contrast, *ATXN3 *has a broad bi- (or tri-) modal distribution of Q-tract lengths [26]. Disease genes can also differ in the number of Q-residues that separate the longest normal from the shortest pathogenic allele. The longest normal *ATN1 *Q-tract is 36Q and the shortest disease allele has 48Q [26,38], while a single residue separates normal (19Q) from pathogenic (20Q) Q-tracts in *CACNA1A *[26,38]. Some disease genes carry non-glutamine interruptions in their Q-tracts, though their lengths are often reported as "repeat lengths" as if they were pure Q-tracts. For example, normal *ATXN1 *has one to three CAT (coding for histidine, H) interruptions near the middle of the Q-tract, but in SCA1 disease alleles the repeat tracts are pure CAGpolyQ [39]. Clearly one must be cautious in making assumptions about common features among polyglutamine expansion disease genes when seeking to identify new disease-associated genes.

At the sequence level, polyglutamine expansion disease genes share several characteristics. They have long uninterrupted CAG tracts [29] and tend to have polymorphic Q-tract lengths [26,36]. Analysis of both genomic DNA and expressed sequence tags have shown that pure CAG-tract length is correlated with Q-tract variance [36,40,41] and interruptions provide stability to repeat tracts [36,37]. Finally, comparisons of orthologous human and rodent genes show that the lengths of disease-associated Q-tracts have a low level of conservation between species compared with those that are not associated with disease [29,42].

The products of the genes causing polyglutamine expansion disorders do not all share a specific function, but the phenotypic overlap of these disorders does suggest some common functions in either their normal or mutated states, or both. As early as 1989, researchers noted the involvement of polyQ-containing genes in transcriptional regulation [43]. This connection spans organisms from yeast to humans [44-48] and known disease-causing genes like *HD*, *TBP *and *ATXN7 *are directly involved in transcription and transcriptional regulation [49-55]. *ATXN1 *and *ATXN2 *are thought to be involved in RNA metabolism [56,57] while *CACNA1A *is the only ion channel gene known to cause a polyglutamine expansion disorder [5]. The normal function of a gene product and the role of the Q-tract in that protein determine the distribution of repeat lengths in the normal population and the threshold for pathogenic expansion for each gene. Therefore, the functions of CAGpolyQ-containing genes must be assessed in conjunction with the normal levels of repeat polymorphism in order to prioritize candidate genes for polyglutamine expansion disorders.

### Summary

Using the human genome reference sequence [58,59] and Ensembl annotated genes [60] we performed a genome-wide computational identification of all candidate genes containing a specific GeMS sequence, CAGpolyQ repeats. We used fragment analysis to assess the CAG-tract lengths of these candidate genes in a large control population. We also applied two methods of analyzing the potential functions of these genes based on the Gene Ontology (GO) system of functional classification [61] in order to identify and visualize the network of functional relationships among the CAGpolyQ-containing genes in the human genome. Using related approaches, Lavoie and colleagues identified polyalanine-containing genes in the human genome and assessed their normal levels of polymorphism [62]. Functional analysis revealed that the majority of polyalanine-containing genes have roles in transcriptional regulation [62].

In characterizing the Q-tract length distributions for 64 CAGpolyQ tracts in 62 genes in the human genome, we find that each Q-tract has a unique distribution of Q-tract lengths. The best predictors of known disease genes were occurrence of a long uninterrupted CAG-tract in their reference genome sequence and high Q-tract length variance in the normal population. Therefore, we used these parameters to identify eight priority candidate genes for polyglutamine expansion disorders. The majority of CAGpolyQ-containing genes are involved in transcriptional regulation and neurogenesis. We provide a visual framework for interpretation of new information on CAGpolyQ gene functions and their biomolecular interactions.

## Results

### Identification of CAGpolyQ-containing Genes

CAGpolyQ repeats were identified on the basis of having tandemly repeated CAG trinucleotides in the sequence within the boundaries of a known gene that had five or more tandem glutamine residues in its peptide sequence (see Methods for detailed description of approach and data sources). Build 33 of the human genome sequence [58] contained 436 CAG trinucleotide repeats in total. Sixty-six of these CAG repeats lay in glutamine-coding sequences in genes including all nine genes in which mutation by expansion of their CAGpolyQ repeat tract is known to cause a neurodegenerative disorder (Table 1).

**Table 1:** Q-tract length variation in genes containing polyglutamine-encoding CAG-type trinucleotide repeats, sorted by Q-tract

**Chromosome Band**	**Gene Name^a^**	**Repeat Sequence from Reference Genome (sense strand)^b^**	**Expected Q-tract Length from Reference Genome^c^**	**N^d^**	**Observed Q-tract Length Min-Max**	**Q-tract Mean**	**Q-tract Variance**
17p13.2	MINK1*	G4N1**G5**	Q4LQ5 (SwP)	162	5 – 5	5.0	0
9q34.11	CIZ1	**G6**	Q6	154	6 – 6	6.0	0
7q36.2	PAXIP1L*	**G7**	Q7	168	7 – 7	7.0	0
11q24.3	PRDM10	**G8**	Q8	172	8 – 8	8.0	0
4q31.1	MAML3a*	**G9**	Q9	156	8 – 8	8.0	0
6p21.1	TFEB	**G6**A1G3	Q10	162	10 – 10	10.0	0
19p13.11	CHERP	**G6**A1G5	Q12	192	12 – 12	12.0	0
12q21.2	PHLDA1	G5A1**G6**A2G1	Q15	212	14 – 14	14.0	0
16p13.3	CREBBP	**G4**A1G3A2G2A1**G4**A1	Q18	158	18 – 18	18.0	0
4q31.1	MAML3b*	G3A1G3A1G1A1**G8**	Q18	166	18 – 18	18.0	0
20q11.22	NCOA6*	G4A4**G8**A2G1A1G2A2G1	Q25	166	25 – 25	25.0	0
Xq13.1	MED12*	G5A1G2A1G1A1G5A1G1A1**G7**N4G6	Q26X4Q6	205	26 – 27	26.0	0
20q13.12	PRKCBP1	**G7**A1	Q8	152	8 – 9	8.0	0.01
15q24.1	ARID3B	**G8**A2G1	Q11	212	11 – 12	11.0	0.01
22q11.21	PCQAPa	G4A1G3N1G5N3G7A1G3N8G3N5G5N1**G8**	Q8FQ5X3Q11X16Q5LQ8	152	11 – 12	11.0	0.01
3p24.3	SATB1	G1A1G3A1G1A1**G7**	Q15	174	15 – 16	15.0	0.01
6q16.2	POU3F2	G3A1G1A1G3A1G2A1**G6**A1G1	Q21	148	21 – 22	21.0	0.01
Xq22.3	FRMPD3	G4A3G4A3G3A3**G7**	Q27 (SwP)	184	26 – 27	27.0	0.01
2q35	TNS	**G9**	Q9	178	9 – 11	9.0	0.02
19p13.12	BRD4	**G5**N1G1N1A1G4A1G1A1	Q5RQEQ8	140	8 – 9	8.0	0.03
12p13.31	PHC1	**G5**A2G1A1G2A1G3	Q15	170	13 – 15	15.0	0.05
9q32	C9orf43	**G6**A1G1	Q8	168	8 – 9	8.1	0.07
1q21.3	TNRC4	A1**G8**A1G4A1	Q15	150	15 – 18	15.0	0.08
17q12	SOCS7	**G7**A1	Q8 (SwP)	134	8 – 9	8.1	0.12
1p31.1	ST6GALNAC5	**G7**A1G4	Q12	150	12 – 14	12.1	0.13
15q26.1	POLG	**G10**A1G2	Q13	164	13 – 15	13.1	0.16
22q13.1	TNRC6B	**G8**	Q8	166	7 – 8	7.8	0.17
12q13.12	MLL2*	G5N1A1G1A1G1A1N1**G7**N1A1G1A1G1A1N1 G2A1G1N1A1G2A1G4N1A2G3A1G1N1A1G2 A1G2N1A1G1A1G1A3G3N1A1G3A1G3	Q5LQ5LQ7LQ5LQ4LQ8LQ7 LQ6LQ10FQ8	184	8 – 11	10.2	0.21
7p14.1	POU6F2	**G10**	Q10	168	6 – 11	10.0	0.22
Xq28	CXorf6	G1A1**G8**A1N92G5A1G4	Q11X92Q10	168	11 – 12	11.6	0.25
12p13.33	DCP1B	**G9**A1	Q10	136	10 – 12	10.5	0.26
17q23.2	VEZF1	**G12**A6	Q13 (through intron)	176	8 – 15	13.1	0.29
22q11.21	PCQAPb	G3A1G2N9A2G1A1**G12**	Q6X9Q16	152	12 – 18	16.1	0.34
3p14.1	MAGI1	G5A1G3A1**G10**	Q20	168	16 – 21	20.3	0.36
4q21.21	BMP2K	G8A1G1A1G4A1G1A1**G9**	Q27	148	23 – 28	26.9	0.36
16q22.1	NFAT5*	**G5**A1G3A1G3A3G1	Q17	168	11 – 19	17.0	0.37
12p13.31	ZNF384	**G14**A1G1	Q16	214	11 – 20	15.2	0.47
22q12.1	MN1*	A1**G9**A1G6A1G1A1G1A1G6	Q28	180	26 – 30	28.7	0.53
12q24.33	EP400	G6A2**G14**A1G4A1G1	Q29	158	28 – 31	28.8	0.53
12q23.2	ASCL1	**G12**	Q12	148	9 – 15	12.3	0.65
6q25.3	ARID1B	**G7**A1G7A1G1A1	Q18	152	16 – 23	17.7	0.69
11q21	MAML2	G1A1G2A1**G13**A1G5A1G1A1G1A1G1A1G2N 5A2G1A1G3N5A1G5A2G5A3G1A2G6A2	Q31X5Q7X5Q27 (through intron)	168	27 – 31	28.3	0.75
12q24.12	**ATXN2**	**G13**A1G9	Q23	124	17 – 27	22.2	0.79
9p24.3	SMARCA2	G1A2G3A1**G13**A1G2	Q23	130	18 – 24	22.7	0.79
20q13.12	NCOA3	G6A1**G9**A1G1A1G1A1G1A1G2A1G2A1	Q29	150	26 – 31	28.4	0.80
17p11.2	RAI1	**G13**A1	Q14	184	11 – 17	14.6	0.84
7q31.1	FOXP2*	G4A1G4A2G2A2G3A5G2A2**G5**A1**G5**A1G1	Q40	100	34 – 40	39.8	0.85
3p14.1	**ATXN7**	**G10**	Q10	184	7 – 14	10.4	0.89
19q13.2	NUMBL	G6A1G1A1**G7**A1G2A1	Q20	156	18 – 20	18.7	0.93
12q24.31	NCOR2	G3A2**G12**	Q16 (through intron)	172	13 – 20	16.9	0.95
15q26.3	MEF2A	**G11**	Q11	174	8 – 16	10.2	1.13
14q24.3	C14orf4	A1G1A1G1A1G6A1**G10**A2G1	Q25 (through intron)	150	20 – 31	23.4	1.17
3q13.2	KIAA2018	**G11**A1G1A4	Q14 (through intron)	150	11 – 16	12.6	1.44
1q21.3	DENND4B	A1G5A1**G9**	Q16	156	13 – 17	15.2	2.04
6p22.3	**ATXN1**	G12T1G1T1**G14**	Q12HQHQ14	130	11 – 21	14.6	2.23
6q27	**TBP**	G3A3G8A1G1A1**G19**A1G1	Q38	158	30 – 41	36.9	2.26
19p13.3	**CACNA1A**	**G13**	Q13	112	7 – 16	12.1	2.42
16p12.1	TNRC6A	**G4**A1G3	Q8	166	4 – 8	7.2	2.50
6p21.1	RUNX2	A1G3A1G4A1**G6**A1**G6**	Q23	100	18 – 30	22.5	3.04
16q22.1	THAP11	G3A1G5A1G2A1G5A1**G10**	Q29	170	18 – 30	28.5	3.12
1q22	KCNN3	G7A1G4N25**G14**	Q12X25Q14	170	15 – 25	20.3	3.98
4p16.3	**HD**^e^	**G19**A1G1	Q21	252	9 – 33	17.2	7.18
Xq12	**AR**	**G22**A1N5G6	Q23X5Q6	180	14 – 33	23.7	9.34
12p13.31	**ATN1**	G1A1G1A1**G15**	Q19	168	11 – 27	17.6	11.6
14q32.12	**ATXN3**	G2A1N1G1A1**G8**	Q3KQ10	168	10 – 27	17.8	29.2
2q37.1	TNRC15	**G6**	Q6	n.d.	n.d.	n.d.	n.d.

^a^Boldface   text marks a gene known to cause disease by expansion of a   polyglutamine-encoding CAG trinucleotide repeat. 'a' and 'b' after MAML3   and PCQAP denote two targets within these genes. Genes marked with an   asterisk (*) contain an additional repeat target that was not screened   in this study.

^b^G denotes   "CAG", A denotes "CAA" and N denotes a non-glutamine codon, each   followed by the number of tandem repeats of that codon. Boldface text   marks the longest uninterrupted CAG-tract.

^c^X indicates a non-glutamine amino acid; SwP indicates peptide sequence obtained from SwissProt record

^d^N denotes number of alleles screened

^e^Data for N, Observed Q-tract Length Min-Max, Q-tract Mean, Q-tract Variance, taken from Andres *et al*. (26)

### Distributions of Q-tract Lengths

Using PCR amplification and ABI fragment analysis we established the range of CAGpolyQ tract lengths for 64 targets (in 62 genes) in a set of healthy individuals of mixed ethnic background (Table 1, Additional file 1). We screened at least 130 normal alleles for each target (mean 162), including X-linked genes, giving us 99% confidence that 95% of the whole population lie between the minimum and maximum values in our sample (95% tolerance; see Methods), with the exception of four targets for which we screened slightly fewer alleles due to technical limitations: *ATXN2 *and *CACNA1A *(94% tolerance), *FOXP2 *and *RUNX2 *(93% tolerance). Table 1 summarizes data for 66 CAGpolyQ repeat targets in 64 genes.

#### Known disease genes have long uninterrupted CAG-tracts and high Q-tract length variances

We sought in our data some hallmark of the nine known disease genes that would allow us to prioritize candidates among the 54 genes not yet associated with CAGpolyQ expansion disorders. Sorting CAGpolyQ repeats by increasing Q-tract length variance (Table 1) clustered disease genes in the top one third of 64 targets. Known disease gene Q-tract length variances ranged from 0.79 (*ATXN2*) to 29.2 (*ATXN3*). The highest Q-tract length variances of all targets were observed in four known disease genes: *ATXN3*, *ATN1*, *AR *and *HD*. The least polymorphic disease target, *ATXN2*, is distinguished from other disease genes by its previously documented tight distribution of Q-tract lengths [26].

Q-tracts are made up of lengths of CAG codons that can be pure or interspersed with one or more CAA codons. Length polymorphism tends to occur within CAG-tracts. Sorting CAGpolyQ repeats by the length of their longest uninterrupted CAG-tract in the reference genome clustered disease genes in the top half of 64 targets. This was increased to the top one third if *ATXN3 *was excluded due to its reference genome sequence reflecting the low mode of a bimodal distribution of repeat tract lengths (see graph in Additional file 2). Disease gene CAG-tract lengths ranged from 10 (*ATXN7*) to 22 (*AR*) and the longest uninterrupted CAG-tracts of all targets occurred in four disease genes: *AR *(22 CAG), *HD *(19 CAG), *TBP *(19 CAG) and *ATN1 *(15 CAG).

**Figure 1:**
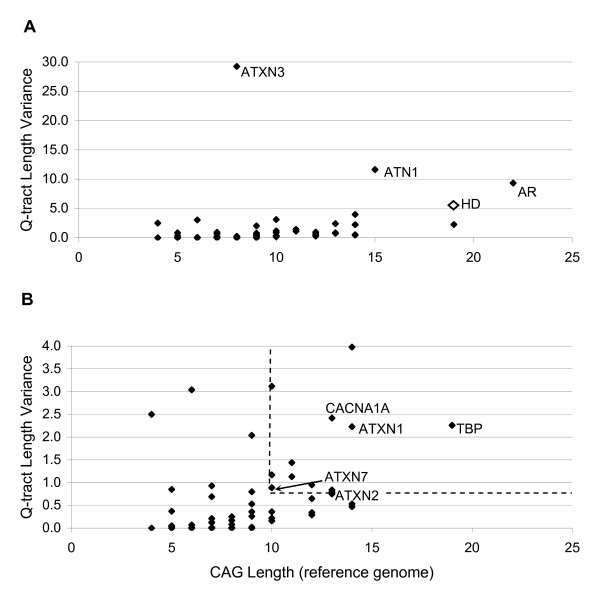
**Relationship between length of longest uninterrupted CAG-tract and Q-tract length variance**. (A) All targets. *HD *Q-tract length variance from Andres *et al*. [26]. Correlation = 0.62, not including *ATXN3*. (B) Higher resolution view of targets with Q-tract length variance < 4.0. Dashed lines at 10 CAG and 0.79 variance represent the cutoff for identifying candidate genes for polyglutamine expansion disorders. See text for list of genes falling in this area.

The length of the longest uninterrupted CAG-tract in the reference genome for each target (*e.g*. CAG_13_CAA_1_CAG_9 _has CAG-tract length of 13; see Table 1) was positively correlated with its level of polymorphism (correlation = 0.62, *ATXN3 *excluded; Figure 1). Given this association between long CAG-tracts and high Q-tract length variance, we divided all targets in two groups at the median CAG length of eight and tested the null hypothesis that variances were equal in the two groups. Q-tract length variances were indeed higher with longer CAG-tracts (p = 0.002, 1-tailed heteroscedastic t-test).

Mean or maximum Q-tract length failed to yield any significant clustering of disease genes, and mean Q-tract length was only very weakly correlated with Q-tract length variance (correlation = 0.12). Underlying this relationship is the fairly weak correlation of uninterrupted CAG-tract length with mean Q-tract length (0.31, *ATXN3 *excluded). Mixtures of CAG and CAA codons making up the Q-tract account for this. One telling example is *FOXP2 *which had the longest mean and maximum Q-tract lengths but relatively little variance in Q-tract length. In fact, *FOXP2 *had the second-shortest uninterrupted CAG-tract of all 66 targets. Based on our analysis, this low level of polymorphism is predicted by the short pure CAG repeat length.

Sorting targets according to other parameters also failed to yield any significant clustering of disease genes. These included sorting by the proportion of alleles with Q-tract lengths longer than mean + 1 SD, and by repeat purity, which was a combined measure of both the length of the longest uninterrupted CAG-tract and the total Q-tract length.

#### Priority candidates for polyglutamine expansion disorders

A plot of CAG length versus Q-tract length variance for each target allowed us to identify eight genes as priority candidates for polyglutamine expansion disorders (Figure 1). We selected genes that had uninterrupted CAG-tracts equal to or longer than 10 CAG (the shortest uninterrupted CAG-tract in a known disease gene, *ATXN7*) and had Q-tract length variance equal to or higher than 0.79 (the lowest Q-tract variance in a known disease gene, *ATXN2*). All eight priority candidates: *C14orf4*, *KCNN3*, *KIAA2018*, *MEF2A*, *NCOR2*, *RAI1*, *SMARCA2*, and *THAP11 *are expressed in normal brain [63-66]. This list is not meant to be exhaustive, but rather a list of the top eight genes prioritized according to two hallmarks of known disease genes.

#### Twelve invariant CAGpolyQ repeats have short CAG-tracts

In this set of 64 CAGpolyQ repeats, having at least four tandem CAG codons coding for five tandem glutamine residues, mean Q-tract length ranged from five to 39.8 (Table 1). Twelve repeats in eleven genes, including CREB-binding protein (*CREBBP*) for example, had no changes in Q-tract length in as many as 212 alleles tested. An additional six repeats were essentially invariant with only one out of as many as 184 alleles differing in length by one Q-residue (Table 1). The twelve invariant repeats had uninterrupted CAG-tracts from four to nine repeat units long but had mean Q-tract lengths evenly distributed from five to 26 residues (Table 1). Thus, a lack of polymorphism was restricted to relatively short pure CAG-tracts but their Q-tract lengths varied widely. This again emphasizes the utility of using pure CAG-tract length rather than Q-tract length in assessments of length polymorphism.

#### Each CAGpolyQ repeat has a unique distribution of Q-tract lengths

**Figure 2:**
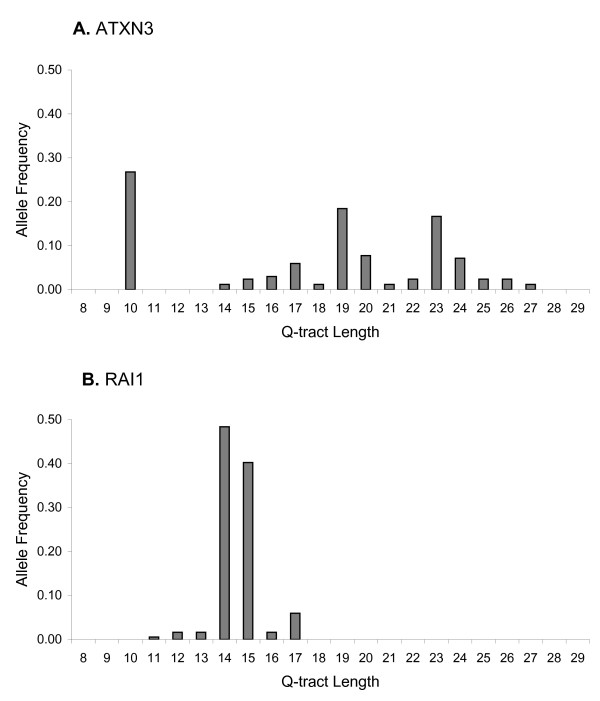
**Example distributions of normal Q-tract lengths**. (A) ATXN3, ataxin 3 (B) RAI1, retinoic acid receptor 1.

The two allele frequency distributions of Q-tract lengths in Figure 2 provide examples of the 64 CAGpolyQ repeats we analyzed. *ATXN3 *had a unique bi- or tri-modal distribution that is virtually identical to published data [26]. *RAI1*, a priority candidate disease gene with a long CAG-tract and relatively high Q-tract variance, had a simpler distribution that is consistent with the published Q-tract length range [62]. The 64 plots of allele frequency distributions of Q-tract lengths for each CAGpolyQ repeat illustrate clearly that there is no single pattern that is typical of Q-tract length distributions across the human genome (Additional file 2).

### Functional classification of CAGpolyQ-containing genes

Browsing descriptions associated with the 64 CAGpolyQ genes suggested an over-representation of genes involved in transcriptional processes and genes involved in chromatin architecture, and thyroid hormone receptor binding. We assessed these and other observations using GO-based classification of these genes to determine whether specific functional categories are statistically overrepresented, to visualize the network of functional relationships among CAGpolyQ-containing genes, and to determine whether priority candidates for polyglutamine expansion are associated with one or more specific GO terms.

#### GO over-representation analysis

We used GoMiner [67] to look for statistical over-representation of CAGpolyQ genes in GO terms in the top four levels of the three GO categories: biological process, molecular function, and cellular component. GO term descriptions can be viewed at the Gene Ontology website [68]. GoMiner contained gene name-GO term annotations for 56 of our 64 genes against a background of 13,598 HGNC genes. Genes without GO term assignments at the time of this analysis were: *C14orf4*, *C9orf43*, *CXorf6*, *DENND4B*, *FRMPD3*, *KIAA2018*, *TNRC15 *and *TNS*. Our null hypothesis was that the genes of interest would be distributed among the chosen GO terms in the same proportions as the background set. GO terms with p-values below the significance threshold (p = 0.05) were considered to be over-represented among CAGpolyQ genes. In negative control experiments (see Methods) we found no over-representation in GO terms under molecular function in 100 replicates. Under biological process, three out of 100 replicates each had one over-represented GO term. Under cellular component, one out of 100 replicates had one over-represented GO term and one out of 100 replicates had two over-represented GO terms.

Over-representation analysis confirmed these 56 CAGpolyQ genes' functional association with transcription and revealed some specific details. There were six significant GO terms under molecular function (Table 2). These included 13.4-fold over-representation of transcription coactivator activity, which is a child term of the 8.8-fold over-represented transcription cofactor activity. CAGpolyQ transcriptional coactivators on our gene list include: *ARID1B*, *CREBBP*, *MAML2*, *MAML3*, *MED12*, *MEF2A*, *NCOA3*, *NCOA6*, and *SMARCA2*. Transcription factor binding was 8.3-fold over-represented, including the transcription coactivator genes above, as well as *HD*, *NCOR2 *and *TBP*. Half of the 56 genes bind DNA. There were five significant GO terms under biological process (Table 2), with the most specific, positive regulation of metabolism, 6.5-fold over-represented (*MAML2*, *CREBBP*, *RUNX2*, *ARID1B*, *NCOA6*, *NFAT5*, and *MAML3*). There were seven significant GO terms under cellular component (Table 2), with nucleoplasm 4.1-fold over-represented. Genes in over-represented GO categories are listed in Additional file 3 (Biological Process), Additional file 4 (Molecular Function) and Additional file 5 (Cellular Component).

**Table 2:** Functional classification of CAGpolyQ genes: Gene Ontology over-representation analysis.

**Gene Ontology term (levels) GO ID**	**Candidate genes in GO term**	Fold* Enrichment
**Biological Process**		
regulation of biological process (**1**) GO:0050789	37	2.3
regulation of physiological process (**2**) GO:0050791	36	2.5
regulation of metabolism (**3**) GO:0019222	29	3.0
positive regulation of metabolism (**4**) GO:0009893	7	6.5
nucleobase, nucleoside, nucleotide and nucleic acid		
metabolism (4) GO:0006139	34	2.5
**Molecular Function**		
transcription regulator activity (**1**) GO:0030528	24	4.0
transcription cofactor activity (**2,**4) GO:0003712	11	8.8
transcription coactivator activity (**3**,5) GO:0003713	9	13.4
nucleic acid binding (2) GO:0003676	35	2.8
DNA binding (3) GO:0003677	28	3.1
transcription factor binding (3) GO:0008134	12	8.3
**Cellular Component**		
organelle (**1**) GO:0043226	43	1.7
membrane-bound organelle (2) GO:0043227	43	1.9
intracellular (2) GO:0005622	47	1.5
intracellular organelle (**2,**3) GO:0043229	43	1.7
intracellular membrane-bound organelle (**3**,4) GO:0043231	43	1.9
nucleus (3,**4**,5) GO:0005634	41	2.7
nucleoplasm (4,**5,**6) GO:0005654	11	4.1

All levels for each GO term are indicated, with boldface indicating one path through the GO

*p < 0.00004 for all GO terms listed except nucleoplasm, p = 0.0001.

#### Shared GO-term analysis

To delve deeper into the possible functional relationships among genes containing CAGpolyQ repeats, we developed a method for quantitative comparison of GO terms annotated to each gene product, based on the structure of the GO graph (AMM, SLB, BFFO, manuscript in preparation). Briefly, given a pair of genes, their GO term annotations, and a comparison scoring function for GO terms, we calculated similarity scores for every pair of GO terms for that pair of genes. GO term pairs scoring above a threshold were used to construct a graph where each node represents a gene and weighted edges between nodes represent pairs of GO term annotations and their scores. Genes were grouped by a simple visual clustering algorithm that assigns shorter lengths to edges with higher weights (i.e. more similar shared GO terms). Because a gene may have multiple shared GO terms with other genes, this method allowed us to cluster the functions of genes that share terms on different branches and at different levels of the gene ontology. Related functions go unnoticed without this clustering.

**Figure 3:**
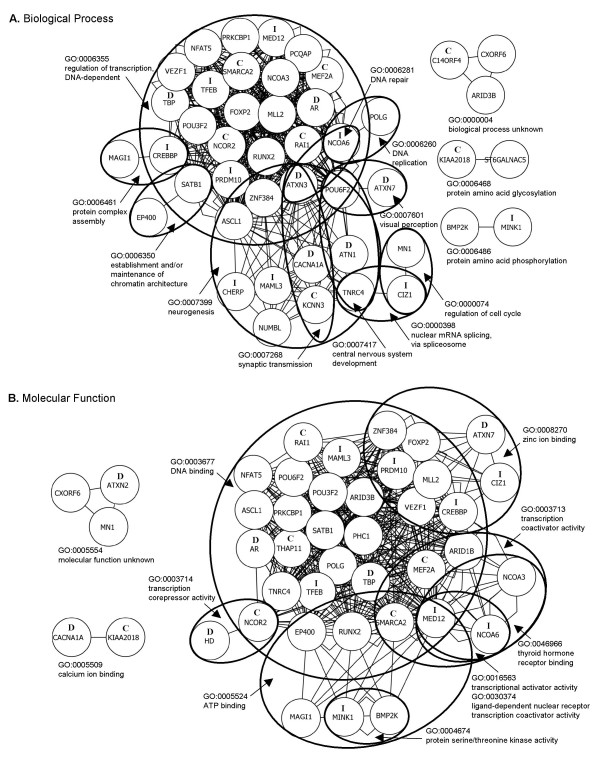
Functional classification of CAGpolyQ genes: shared Gene Ontology term analysis. Known disease genes are marked with a '**D**', candidate disease genes are marked with a '**C**' and genes with invariant Q-tracts (Table 1) are marked with an '**I**'. Clusters of genes are labeled with the GO terms that best described each cluster. GO terms shared by gene pairs are listed in Additional file 7 and Additional file 8. Genes not represented in a graph either had no annotation under that GO namespace or did not share a GO term with a score above the 99^th ^percentile. (A) Biological process. Genes not represented: *ARID1B*, *ATXN1*, *ATXN2*, *BRD4*, *C9ORF43*, *DCP1B*, *HD*, *DENND4B*, *FRMPD3*, *MAML2*, *PAXIP1L*, *PHC1*, *PHLDA1*, *SOCS7*, *THAP11*, *TNRC15*, *TNRC6A*, *TNRC6B *and *TNS*. (B) Molecular function. Genes not represented: *ATN1*, *ATXN1*, *ATXN3*, *BRD4*, *C14ORF4*, *C9ORF43*, *CHERP*, *DCP1B*, *KCNN3*, *DENND4B*, *FRMPD3*, *MAML2*, *NUMBL*, *PAXIP1L*, *PCQAP*, *PHLDA1*, *SOCS7*, *ST6GALNAC5*, *TNRC15*, *TNRC6A*, *TNRC6B *and *TNS*.

Only seven gene pairs scored above the cutoff (estimated 99^th ^percentile; described in Methods) for the cellular component category (Additional file 6) so we did not consider this category further. There were 544 gene pairs with scores above the cutoff in the biological process category, representing 45 genes. There were 503 pairs among 42 genes in the molecular function category. The functional relationships among these CAGpolyQ genes are illustrated in Figure 3. GO terms and the genes that share them are listed in Additional file 7 (Biological Process), Additional file 8 (Molecular Function) and Additional file 6 (Cellular Component).

Based on our analysis of relationships among GO terms shared by two or more genes, CAGpolyQ genes in the human genome clustered primarily under two major biological processes: DNA dependent regulation of transcription, and neurogenesis (Figure 3A). Other processes included establishment and/or maintenance of chromatin architecture and post-translational modifications. Since there were few functional clusters, it was not surprising that all but one known disease gene and most priority candidate genes were involved in DNA dependent regulation of transcription and in neurogenesis (Figure 3A). *ATXN7*, the one disease gene excluded from the cluster involved in DNA dependent regulation of transcription, was recently shown to be an integral component of the TFTC (TATA-binding protein-free TAF-containing) complex and the STAGA (SPT3/TAF9/GCN5 acetyltransferase) complex involved in transcriptional regulation [52-54]. Consistent with their predominant classification in DNA dependent regulation of transcription, DNA binding was the primary shared molecular function among these 64 genes (Figure 3B). Known disease genes were involved in DNA, calcium and zinc binding and HD was classified as having transcription corepressor activity (Figure 3B). All but one priority candidate gene had DNA binding activity according to current GO annotations. CAGpolyQ genes with invariant Q-tract lengths were not limited to any one biological process or molecular function.

## Discussion

Our findings build on previous work indicating that uninterrupted CAG-tract length, not the Q-tract length encoded by CAG plus CAA codons, influences the degree of polymorphism of a Q-tract. Uninterrupted CAG-tract length and Q-tract length variance are the most useful parameters in characterizing known disease genes and identifying candidate genes for expansion disorders. At one extreme, zero variance CAGpolyQ repeats – those that do not tolerate changes in Q-tract length – can likely be excluded as candidates for polyglutamine expansion disorders. The shapes of Q-tract length distributions differed widely between various loci across the genome. Thus, the data presented here for allele length distributions for 64 Q-tracts in 62 genes with detailed conditions for their screening, will be invaluable for identifying putative expansion mutations in candidate genes not yet associated with CAGpolyQ-type neurodegenerative disorders. All nine known polyglutamine expansion disorder genes are involved in DNA-dependent regulation of transcription or in neurogenesis, as are all of the well-characterized priority candidate genes identified in this study.

Many groups have published lists of CAGpolyQ-containing genes identified using classical [15,17-20] or computational methods [21-24]. The content of each computationally-derived list differs slightly depending on the repeat detection algorithms and gene data sets used but they are largely the same. Tandem Repeat Finder, used in this study under default parameters, is not guaranteed to find all CAGpolyQ repeats, but it is likely that the vast majority of long repeats were found. Our approach is validated by its detection of all nine genes known to cause diseases by expansion of CAGpolyQ repeats. This study of the normal levels of polymorphism of human CAGpolyQ repeats is the most exhaustive conducted to date.

Our allele frequency distributions match those published for known disease genes *AR *[69], *ATN1 *[2,3,26,70], *ATXN1 *[26], *ATXN2 *[26], *ATXN3 *[26,70], *ATXN7 *[70,71], *CACNA1A *[26,70], and *TBP *[70,72]. The same is true for CAGpolyQ repeats in other genes whose Q-tract lengths have been found to be invariant like *CREBBP *[25] and *MED12 *[19], moderately polymorphic *FOXP2 *[73], *NCOA3 *[25,26,74], *POLG *[75], *RAI1 *[76], *SMARCA2 *[28] or highly polymorphic *THAP11 *[28] and *KCNN3 *[26,77]. Differences in apparent repeat lengths between this study and published data for *ATXN1 *[26,70] and *ATXN3 *[26,70] exist because we report repeat lengths based on the longest pure Q-tract while Andres *et al*. [26] and Juvonen *et al*. [70] report "repeat lengths" that contain non-glutamine amino acids. For *ATN1*, the shape of our distribution matches published data but our distribution is increased by two to four glutamine residues.

Among our eight priority candidate genes some features are already known. CAG length variation in *RAI1 *is responsible for 4.1% of age of onset variability in SCA2 [76]. Huang *et al*. [42] identified RAI1 (called RAI2 in that paper) and NCOA3 as candidate disease genes by virtue of their long CAG tracts and the fact that their mouse and rat orthologues had Q-tracts less than half the size of the human repeats. In our study, NCOA3 lay just below the threshold for priority candidate disease genes, with nine CAG while priority candidates had ten CAG. *KCNN3 *CAG-tract length differences have been associated with anorexia [78] and with schizophrenia and bipolar disorder but these associations are controversial [79]. *SMARCA2 *and *THAP11 *were previously identified as candidates by Pandey [28] based on their relatively long uninterrupted CAG-tracts. Four genes identified by Huang *et al*. [42] as candidate genes of interest fell far below our threshold of Q-tract variance so we do not consider them to be priority expansion disease candidates. These were DCP1B, MAML3 (called TNRC3 in Huang *et al*.), POLG (called NFYC in Huang *et al*.) and POU6F2 (called RPF-1 in Huang *et al*.).

Q-tract lengths for many genes do not have a normal distribution and differ widely between loci, as previously observed [27,36]. Even different disease genes have very different Q-tract length distribution shapes with different minima and maxima in normal populations and different minimum disease allele lengths so it is critical to characterize each distribution without making generalizations between loci. A gene containing more than one CAGpolyQ repeat can have two invariant repeats (*MAML3*) or a combination of invariant and variant repeats (*PCQAP*). Orthologous repeats in human and mouse genomes can have very different levels of polymorphism: human *VEZF1 *has a polymorphic Q-tract (this study) while the corresponding Q-tract in its mouse orthologue is invariant [80].

### Long pure repeats expand

Alba and colleagues [29,30,81] have clearly shown that, with respect to evolutionary processes, there are two classes of Q-tracts in human proteins: those whose lengths are conserved between human and mouse orthologues, and those whose lengths differ. Length-conserved polyQ repeats tend to be encoded by mixtures of CAG and CAA codons and are likely to be restricted in length by purifying selection. PolyQ repeats whose lengths vary between human and mouse tend to be encoded by longer pure CAG-tracts that evolve nearly neutrally [29,30,81]. Our data on Q-tract polymorphism within a normal human population corroborates their between species data and builds on previous work, with longer pure CAG-tracts having higher Q-tract length variance and invariant CAGpolyQ repeats having relatively short pure CAG-tracts [40,41]. Again, the extremes reinforce the rules; *FOXP2 *with a short 5-CAG repeat has the longest mean Q-tract length of all candidate genes but a low level of polymorphism.

Correlation of uninterrupted CAG length with Q-tract length variance is consistent with work on dinucleotide repeats [82] and on tetra- and penta-nucleotide repeats [83]. For all of these, the level of polymorphism increases with the number of pure repeats, and non-polymorphic repeats have the shortest pure repeat tracts. Similarly, in the *HD *gene, as CAG repeat number increases, there is a significant increase in the frequency of expansion mutations and the mean number of repeats added per expansion [34].

Pure CAG length is not the only factor determining repeat instability. An in-frame interruption in a CAG-tract has a stabilizing influence over and above that of reducing the pure CAG-tract length. In yeast, dinucleotide repeats with a single dinucleotide interruption in the middle of the tract are five times more stable than a pure repeat of the same length [84]. SCA1 disease alleles of the *ATXN1 *gene all contain uninterrupted tracts of CAG repeats while virtually all normal alleles have one to three CAT (coding for histidine) interruptions in the middle of the Q-tract [39]. Other factors underlying repeat instability include different repair mechanisms [32], flanking sequence elements [85,86], CpG methylation, and nucleosome and replication origin positioning [86-88].

Rozanska *et al*. [89] recently published a large study that complements our results, analyzing repeat lengths and interruption patterns in a normal Polish population. They determined that the length of uninterrupted repeat tract in the most frequent allele for a locus is correlated with the degree of length polymorphism for that tract, and provide further evidence for a stabilizing effect of repeat interruptions. Trinucleotide repeat expansion disease genes were found to have a higher proportion of long repeat alleles than those not associated with disease [89].

### Inferences about repeat lengths and disease prevalence

Lack of detailed reporting of repeat sequence lengths in disease genes, such as Q-tract lengths in *ATXN1 *and *ATXN3 *are a potential source of confusion in the literature and highlight the difficulties in comparing Q-tract length distributions for the same genes from different publications. The amino acid sequence of the most common normal *ATXN1 *repeat tract is Q_12_H_1_Q_1_H_1_Q_14 _[37] but it is frequently reported as 29 "repeats" and the *ATXN3 *repeat tract, Q_3_K_1_Q_10_, is reported as 14 "repeats" [26]. Non-glutamine interruptions in a Q-tract are critical to phenotype, so it is misleading to report these as "Q repeats" or "CAG repeats". For this reason, we reported all target Q-tract lengths based on the longest uninterrupted Q-tract (encoded by CAG/CAA) in the reference genome (Table 1, Additional file 2).

Measuring Q-tract lengths in affected individuals enables identification of putative repeat expansions outside the normal range, but more in depth characterization requires precise determination of the underlying amino acid and nucleotide sequences of individual alleles. Characterization of each allele at the nucleotide sequence level in addition to the normal (wild-type) Q-tract length distribution will be critical in better identifying candidate CAGpolyQ genes not yet associated with disease, determining which alleles at a given locus are prone to expansion, and for disease genes, characterizing allele repeat sequences with respect to disease prevalence in a given population [33]. As has been expertly laid out by Sobczak and Krzyzosiak [37] repeat interruption patterns in a given target can differ between populations, even when Q-tract length distributions are similar. Repeat interruption characteristics are not commonly studied, but reporting overall repeat lengths in the absence of repeat interruption patterns may be quite misleading in studies of allele lengths as they relate to disease prevalence in a given population [37,70,90]. Juvonen and colleagues [70] recently reported that the frequencies of large normal alleles at SCA loci were poor predictors of the prevalence of the respective diseases in Finland but Q-tract lengths were assayed without reporting CAG-tract interruption patterns in different alleles. A different picture might be revealed by characterization of repeat interruption patterns at each SCA locus in that population.

### The genotype-phenotype connection

Q-tract length variance is influenced both by specific sequence characteristics and by the specific role of the Q-tract within a protein's structure and function. *AR *provides an excellent example of this balance. The *AR *Q-tract in the reference genome has a very long pure CAG-tract of 22 CAGs, consistent with its high length variance. The CAGpolyQ tract in the AR protein lies in its N-terminal transactivation domain which interacts with the C-terminal ligand binding domain (the N/C interaction). Buchanan *et al*. [69] found no changes in *in vitro *N/C interaction for Q-tract lengths of 16 to 29 but shorter or longer tracts resulted in a significant decrease in N/C interaction. Over 90% of normal alleles fall within the Q16-Q29 range both in this study and in Buchanan's re-examination of published data [69]. Q-tracts in *AR *equal to or longer than 38 glutamines cause the polyglutamine expansion disorder spinal and bulbar muscular atrophy while short Q-tracts are associated with increased risk of prostate cancer [69]. In other genes, Q-tracts with no length variation suggest the presence of strong purifying selection in which a precise Q-tract length is required to maintain a protein's structure or its biomolecular interactions, and its function. Therefore, a length change in a non-variant Q-tract is presumed to be lethal.

### CAGpolyQ Gene Functions

Based on GO overrepresentation and shared-term analysis we find that CAGpolyQ genes are involved, in general, in two major biological processes, DNA dependent regulation of transcription and neurogenesis, and are enriched for transcriptional coactivator and transcription factor binding functions. Subgroups of genes such as known polyglutamine expansion disease genes, priority candidates, or genes containing invariant Q-tracts are not obviously distinguished by association with a particular process or molecular function. Polyglutamine-containing proteins in organisms from yeast to humans have been previously noted to be involved in transcriptional regulation [44-48]. In fact, most eukaryotic repeat containing proteins are involved in transcription or translation or interact directly with DNA, RNA or chromatin, irrespective of the amino acid repeat type [48]. The majority of repeat-containing proteins perform roles in processes that require the assembly of large multiprotein or protein/nucleic acid complexes [48]. Expanded Q-tracts in HD and ATN1 gene products interfere with CREBBP-activated gene transcription via interaction of their Q-rich domains [91,92] and mutant HD targets specific components of the core transcriptional machinery, in a Q-tract length-sensitive manner, to disrupt gene expression in cultured HD cells [55]. We anticipate that continual incorporation into the GO of newly published information about the normal functions of polyglutamine expansion disorder genes will reveal more specific shared functions among them.

## Conclusion

We have characterized the levels of Q-tract length polymorphism in 64 CAGpolyQ repeat tracts in a normal human population, and found a strong positive correlation between uninterrupted CAG-tract length and Q-tract length variance. The best predictors of known disease genes were the occurrence of a long uninterrupted CAG-tract in the reference genome sequence and high Q-tract length variance in the normal population. Using these criteria we identified eight priority candidate genes for polyglutamine expansion disorders based on the presence of pure CAG-tracts longer and Q-tract variances higher than the smallest values in known disease genes. Twelve invariant Q-tracts (in eleven genes) are unlikely to be candidates for polyglutamine expansion disorders. Each CAGpolyQ repeat, including those in known disease genes, has a unique distribution of Q-tract lengths, emphasizing the need to characterize each distribution without making generalizations between loci. This publication makes freely available for the first time the length distributions of virtually all of the CAGpolyQ repeats in the human genome. Using these normal repeat distributions against which pathogenic expansions can be identified, we have begun screening for mutations in individuals clinically diagnosed with SCA or Huntington disease-like disorders who do not have identified mutations within known disease genes.

## Methods

### Selection of candidate genes

Candidate genes were identified on the basis of having a CAG-type simple repeat within the boundaries of a known gene with five or more tandem glutamine residues in the peptide sequence of that gene. To accomplish this, the Simple Repeats table (simpleRepeat.txt.gz) was downloaded from the UCSC genome annotation database [59] for build 33 (April 2003) of the human genome sequence assembly [58] and uploaded into a local MySQL database. The Simple Repeats table contained chromosomal location coordinates of all repeats detected by Tandem Repeat Finder (TRF) software [93] using default parameters. Locations of all the CAG-type repeats in this table were exported to a file using an SQL query to extract all records with the sequences 'CAG', 'AGC', 'CGA', 'CTG', 'GCT' and 'TCG' to accommodate all six potential reading frames of the repeat as they might appear in genomic sequence. This file was used as input to a Perl script that used the Ensembl Perl API [60] version 15_33 to extract all known genes (Ensembl-predicted transcripts that map to species-specific SwissProt, RefSeq or TrEMBL database entries) whose chromosomal coordinates overlapped with the repeat coordinates. For each known gene with a CAG-type repeat, if the Ensembl peptide sequence contained five or more glutamine residues in tandem, that gene was considered a candidate. A minimum glutamine repeat length of five was used since Karlin [94] determined that for a "typical" protein of 400 residues and average composition, a run of an individual amino acid is statistically significant if it is five or more residues long [94].

The candidate gene list was generated from Build 33 of the human genome sequence assembly (April 2003), and the nucleotide/amino acid sequences of each glutamine tract reported in Table 1 were generated from Build 35 (May 2004). Two new candidate genes were identified in the later build (Ensembl known genes data set version 30_35c) that were not part of our study: *MKL1 *and *C14orf43*, and additional CAGpolyQ repeats were detected in nine of our existing candidate genes: *FOXP2*, *MAML3*, *MED12*, *MINK1*, *MLL2*, *MN1*, *NCOA6*, *NFAT5*, *PAXIP1L*. These targets have been denoted by an asterisk in Table 1. Chromosome band was obtained from the UCSC Chromosome band track [95] and may differ slightly from a gene's location listed by the HGNC Database, Genew [14]. Gene names listed are official HGNC gene symbols from the HGNC website [96] (accessed March 13, 2007).

### DNA samples

Control DNA samples (extracted from blood) were from a population of mixed ethnic background with individuals of Western European descent most highly represented (Additional file 1). 48 of these were from the Coriell Cell Repository [97].

### PCR primers and amplification of candidate repeats

Additional file 9 lists primer sequences, annealing temperatures, specific PCR conditions and expected fragment size (from the reference genome) for each repeat target. PCR primers for candidate repeat amplification were designed using Primer3 [98]. Forward primers were 5'-labeled with 5-HEX, 6-FAM or TAMRA fluorescent dyes (Operon) and reverse primers all had a 5'-GTTT "PIG-tail" [99]. PCR amplification was performed with standard Taq polymerase (Invitrogen) or AccuPrime Taq polymerase (Invitrogen) in 96-well plates according to the conditions specified for each target in Additional file 9. PCR products were visualized and quantitated by comparing the signal intensity of a specific volume of PCR product against 4 μl of Low DNA Mass Ladder (Invitrogen) on an agarose gel. The accuracy of this quantitation method was validated against the PicoGreen^® ^dsDNA Quantitation assay (Molecular Probes) [100].

### ABI 3700 fragment analysis and GeneMapper band calling

PCR products for fragment sizing were assembled in 96-well microtiter plates at 0.5 ng/μl in each well, with up to six PCR products multiplexed per well according to their predicted allele sizes and fluorescent labels. One microliter of the multiplexed PCR products was added to 9 μl of either 2% 400 HD [ROX] sizing standard (Applied Biosystems) or 2% 500 [ROX] sizing standard (Applied Biosystems) depending on the estimated sizes of products being analyzed. DNA fragments were separated by capillary electrophoresis using the ABI Prism 3700 DNA Analyzer (Applied Biosystems) with POP-6 polymer (Applied Biosystems). Sizing of the PCR fragments was accomplished using GeneMapper software (v.3.0, Applied Biosystems). Representative alleles from each locus were sequenced to determine the exact correspondence between fragment size and Q-tract length. In all cases (except TNRC15, for which we do not present data), fragment length polymorphism was entirely accounted for by changes in Q-tract length. At least one such sequenced allele was included on every run as a calibrator.

### Data management and analysis

Repeat information, PCR conditions, sample information and analysis results were stored in a MySQL database called GeMSdb (Genomic Mutational Signature sequences database). Data was input into GeMSdb using Perl scripts and through a web interface built with PHP and Apache. Data analysis and graphics were done using PHP.

The Q-tract length of each allele was based on the difference between observed PCR fragment size from a DNA sample and expected PCR fragment size from the reference genome (plus 4 nucleotides from the primer tail). Expected fragment sizes and Q-tract lengths (reference genome Build 35) for every target are listed in Additional file 9. Q-tract length_Exp _below is that of the longest uninterrupted Q-tract in the target. For example, the *ATXN1 *Q-tract (Q_12_H_1_Q_1_H_1_Q_14_) length_Exp_is 14 because the overall repeat region of 29 residues is interrupted by two non-glutamine amino acids.

Q-tract length_Obs _= (Fragment size_Obs _- Fragment size_Exp_)/3 + Q-tract length_Exp_

Repeat purity was calculated as a normalized weighted measure, nWP, combining both the length of the longest uninterrupted CAG-tract (CAG-length) and the total Q-tract length (Q-length) of each repeat. Weighted purity (WP) for each repeat was normalized by dividing by the highest WP among loci, which was 21.04 for *AR*.

nWP = (CAG-length/Q-length)*CAG-length/21.04

### Statistical analysis

Because there was no *a priori *knowledge of the distribution of Q-tract lengths in each gene for the typical control population, we applied the statistics of tolerance levels to determine the number of control alleles that must be screened to distinguish a Q-tract length that occurs in the affected but not unaffected populations with a given level of confidence. Screening 130 control alleles provides us with 99% confidence that 95% of the population of interest lies between the minimum and maximum repeat lengths in our samples [101].

### Gene expression

Candidate genes' expression in brain was determined according to either eVOC controlled vocabularies for gene expression data [63,64] queried through BioMart [65] or according to expression data at the GeneCards website [66] (accessed September 19, 2005).

### Gene functional classification

#### Gene Ontology over-representation analysis

We used GoMiner [67] for GO over-representation analysis down to the fourth level in the ontology. The target and background gene sets were generated as follows. We downloaded 23,913 HGNC gene IDs on June 28, 2005 from the HGNC website [96]. All IDs ending in '~withdrawn' were removed to generate a list of 21,591 IDs used as the 'query gene file' for GoMiner. GoMiner matched 13,598 of these to GO terms. We conducted 100 negative control replicates of this experiment for the three GO categories, each replicate with 56 randomly selected genes out of the 13,598 background gene set. To correct for multiple testing we used a Bonferroni correction to adjust the threshold of significance appropriately. The raw threshold of significance was p = 0.05. Adjusted significance thresholds were: molecular function p = 0.00004; biological process p = 0.00005; cellular component p = 0.00009.

#### Graph-based shared Gene Ontology term analysis

For each pair of genes among our set of 64, the GO terms annotated to each gene were compared and we calculated a graph-based similarity measure (AMM, SLB, BFFO, manuscript in preparation) for all gene pairs. In order to determine significant scores and produce a meaningful subgraph, we bootstrapped an estimate of the score required to be above the 99^th ^percentile for a set of genes of that size (64) from the background set. We randomly drew 1000 replicates from the set of 15,168 Entrez Gene human protein-coding genes and took the mean of the 99^th ^percentile score for each GO namespace (biological process, molecular function and cellular component) as our cut-off value. Pairs of genes with shared GO terms scoring above the cut-off value were visualized using Cytoscape 2.1 [102] with the "organic" arrangement of nodes, which produced a natural set of clusters. The "organic" node arrangement treats edges as springs: the more edges among a group of nodes, the tighter they cluster. The pairwise similarity measure links GO terms via their lowest common ancestor term in the graph. These lowest common ancestor terms are output with each pair of GO terms that are scored, and can be considered as edge labels in the resulting graph. Clusters of genes joined by the same GO term edge labels were manually annotated with those GO terms.

## Abbreviations

CAGpolyQ, polyglutamine-encoding CAG trinucleotide repeat; Q-tract, polyglutamine tract; HD, Huntington disease; SCA, spinocerebellar ataxia; ATN1, atrophin1; AR, androgen receptor; TBP, TATA-binding protein; ATXN, ataxin; GeMS, Genomic Mutational Signature; GO, Gene Ontology; HGNC, Human Gene Nomenclature Committee

## Authors' contributions

SLB, RSD, BRL, BFFO and RAH conceived and designed the experiments. SLB, RSD, CLM, AMM, SJN, SSL, AW, GSY and MMSY performed the experiments. SLB, YH, SJN, CLM and AMM analyzed the data. CLM and YH designed and managed the database. MRH and RAH contributed reagents/materials. SLB wrote the paper and all authors read and approved the final manuscript.

## Supplementary Material

Additional file 1 Ethnic composition of control population. Click here for file

Additional file 2 Allele length distributions in a normal population for 64 polyglutamine-encoding CAG trinucleotide repeat targets (A) – (BL). This multi-page document provides plots of allele frequency distributions. Click here for file

Additional file 3 Genes in over-represented GO terms under Biological Process. For each over-represented GO term and its GO ID, this document lists the CAGpolyQ repeat-containing genes that were annotated with that GO term. Click here for file

Additional file 4 Genes in over-represented GO terms under Molecular Function. For each over-represented GO term and its GO ID, this document lists the CAGpolyQ repeat-containing genes that were annotated with that GO term. Click here for file

Additional file 5 Genes in over-represented GO terms under Cellular Component. For each over-represented GO term and its GO ID, this document lists the CAGpolyQ repeat-containing genes that were annotated with that GO term. Click here for file

Additional file 6 Genes and their shared GO terms under Cellular Component. This document provides GO IDs, their descriptions, and the lists of CAGpolyQ repeat-containing genes that shared these annotations above the 99^th ^percentile cutoff. Click here for file

Additional file 7 Genes and their shared GO terms under Biological Process. This document provides GO IDs, their descriptions, and the lists of CAGpolyQ repeat-containing genes that shared these annotations above the 99^th ^percentile cutoff. Click here for file

Additional file 8 Genes and their shared GO terms under Molecular Function. This document provides GO IDs, their descriptions, and the lists of CAGpolyQ repeat-containing genes that shared these annotations above the 99^th ^percentile cutoff. Click here for file

Additional file 9 Conditions for PCR amplification of CAGpolyQ repeats in 64 CAGpolyQ repeats. This table provides primer sequences, annealing temperatures and expected fragment sizes for screening these repeats. Click here for file
