# Supplementary material for: CAG-encoded polyglutamine length polymorphism in the human genome
Source: BMC Genomics. 2007 May 22;8:126. doi: 10.1186/1471-2164-8-126 (PMC1896166; doi:10.1186/1471-2164-8-126)
Supplement: Additional file 2 — Allele length distributions in a normal population for 64 polyglutamine-encoding CAG trinucleotide repeat targets (A) – (BL). This multi-page document provides plots of allele frequency distributions. [file 1471-2164-8-126-S2.pdf]

Wild-type allele distributions for 64 polyglutamine-encoding CAG trinucleotide repeat targets.

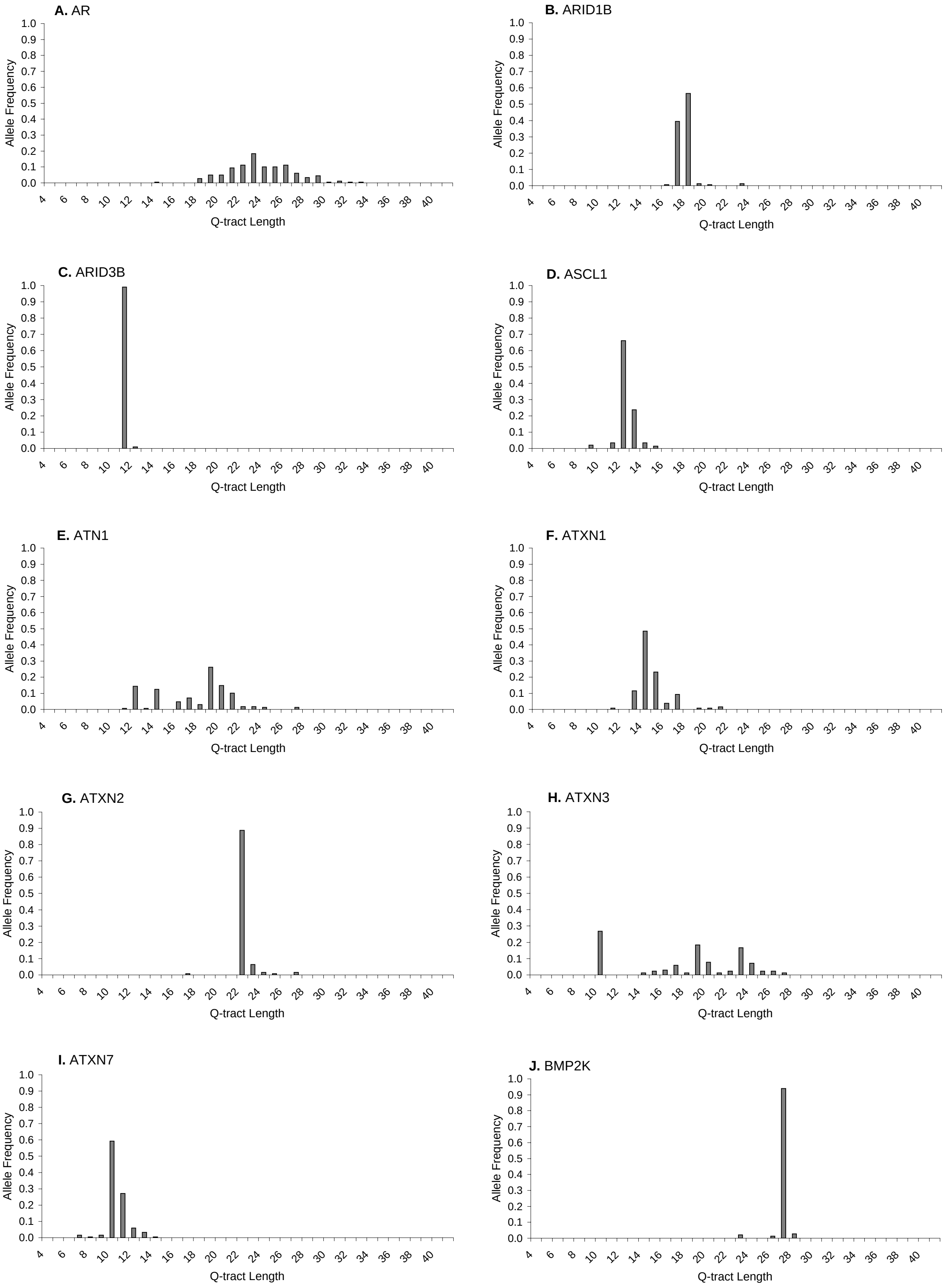

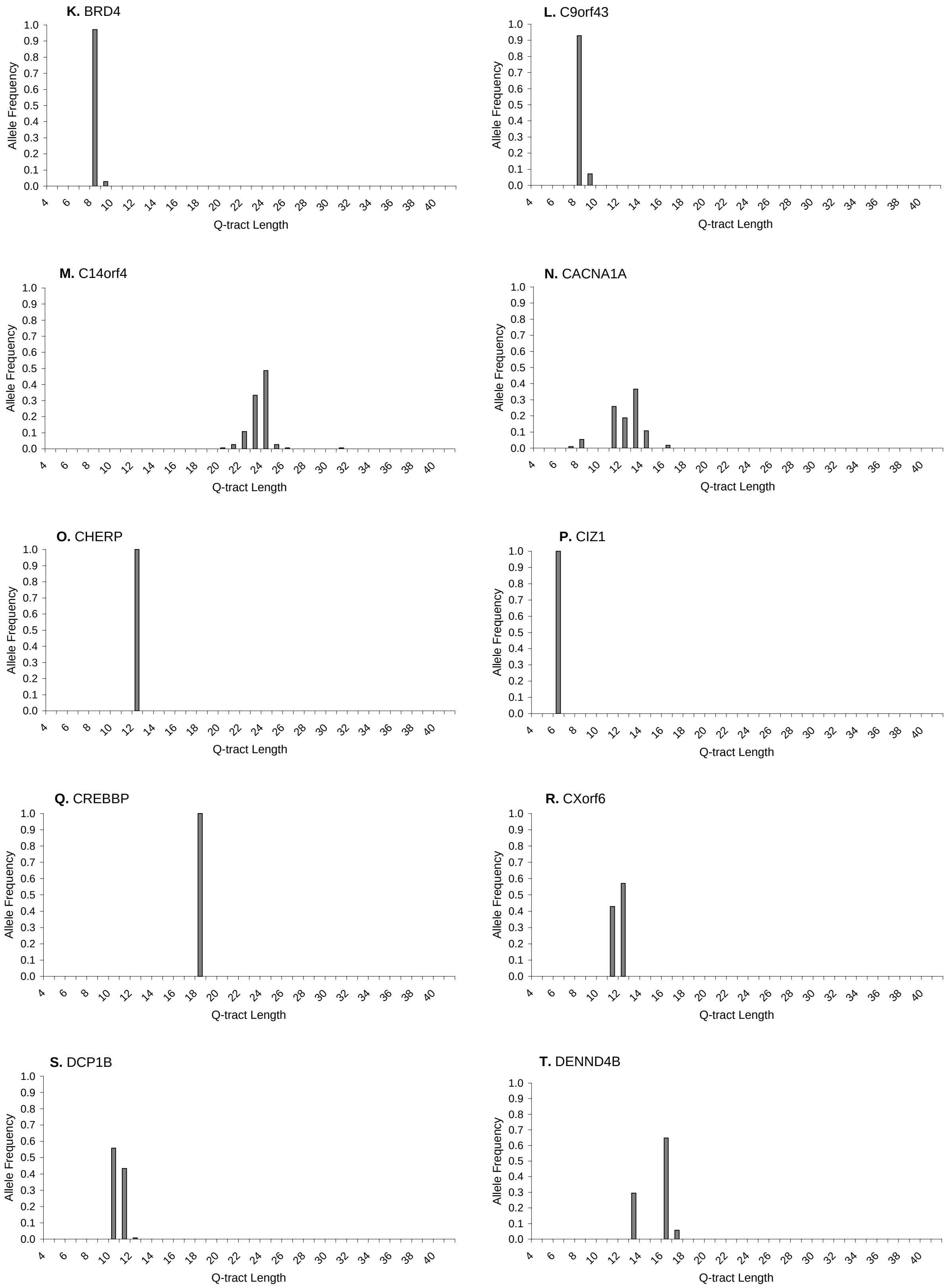
K. BRD4
L. C9orf43
M. C14orf4
N. CACNA1A
O. CHERP
P. CIZ1
Q. CREBBP
R. CXorf6
S. DCP1B
T. DENND4B
Allele Frequency
Q-tract Length

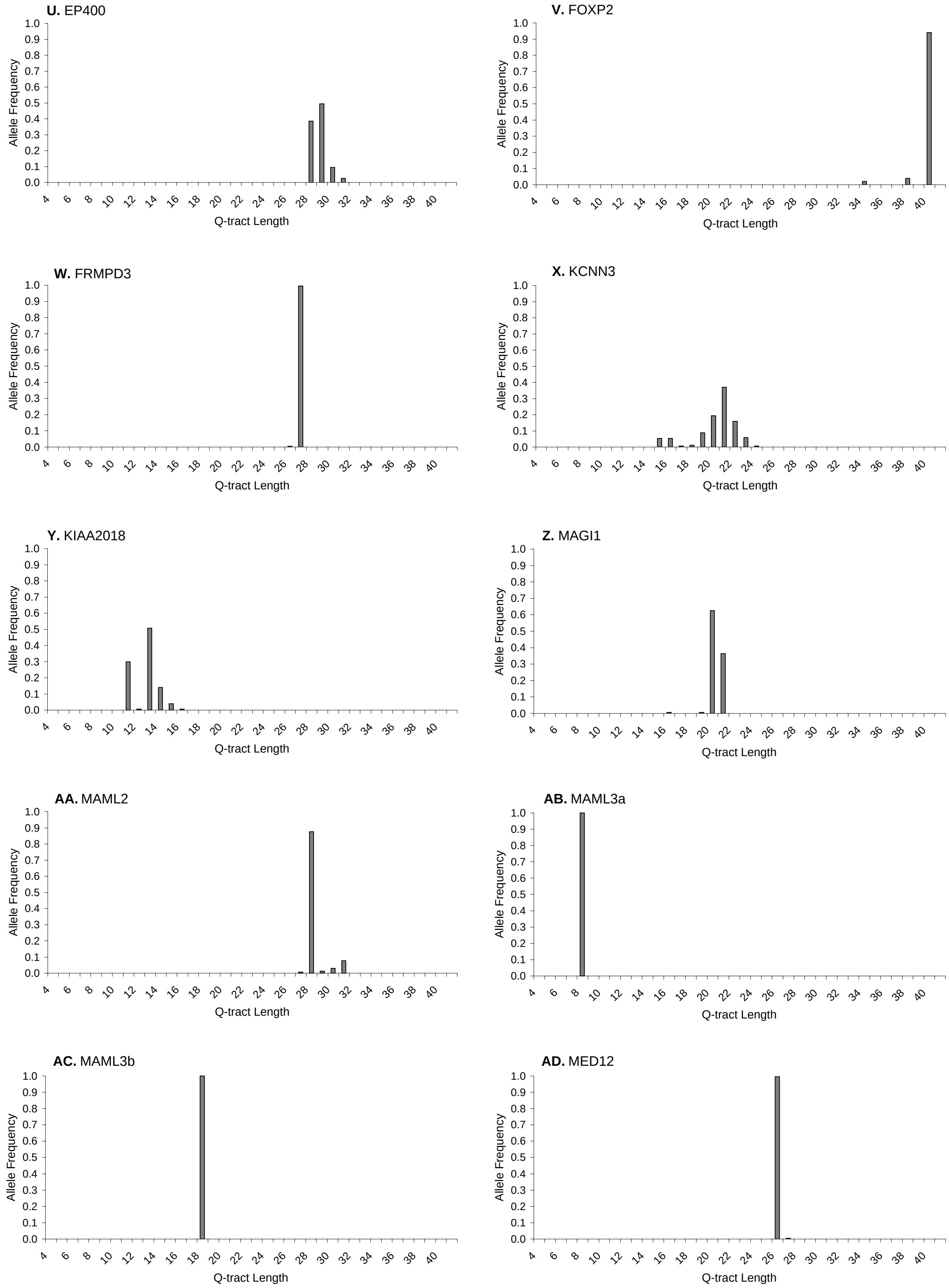
U. EP400
V. FOXP2
W. FRMPD3
X. KCNN3
Y. KIAA2018
Z. MAGI1
AA. MAML2
AB. MAML3a
AC. MAML3b
AD. MED12
Allele Frequency
Q-tract Length

AE. MEF2A

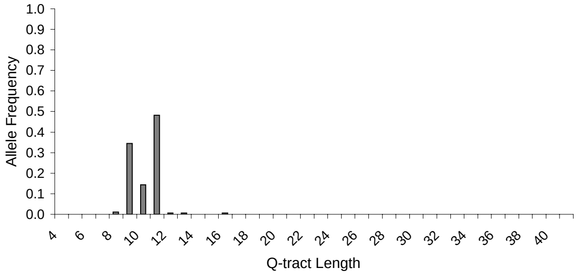

AF. MINK1

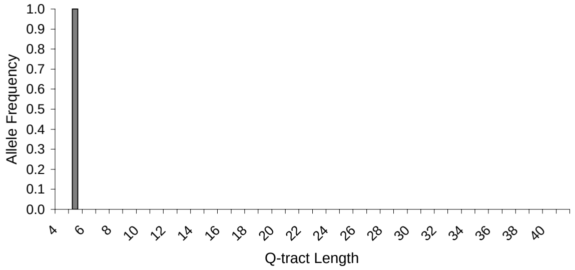

AG. MLL2

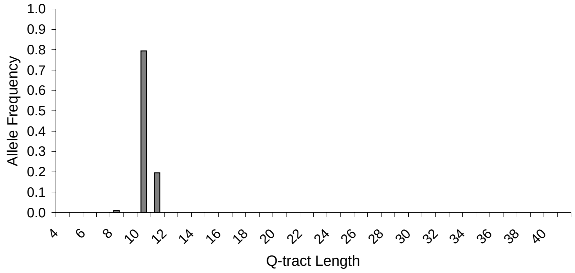

AH. MN1

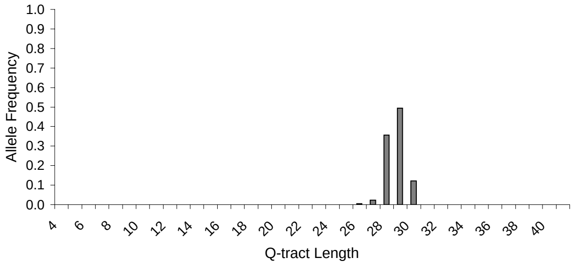

AI. NCOA3

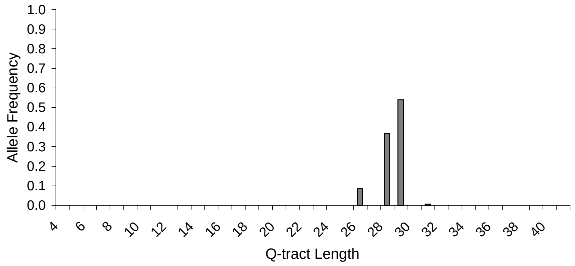

AJ. NCOA6

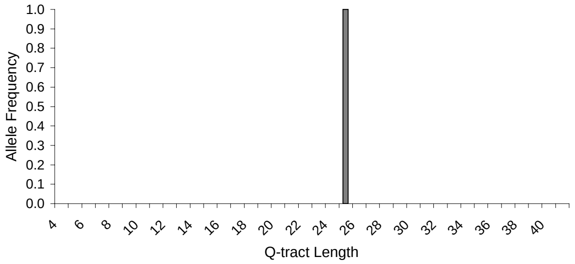

AK. NCOR2

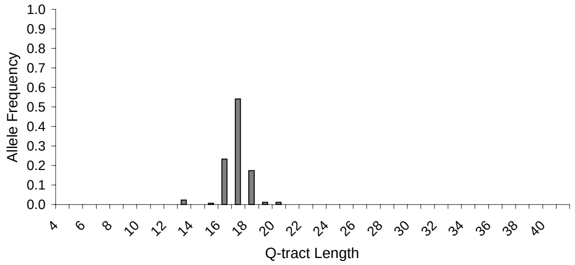

AL. NFAT5

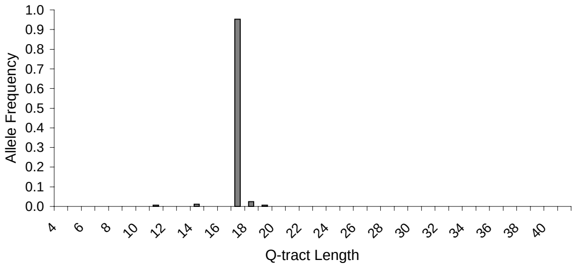

AM. NUMBL

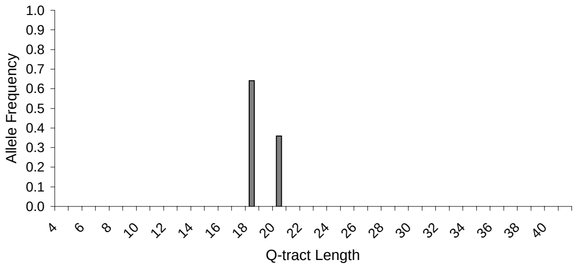

AN. PAXIP1L

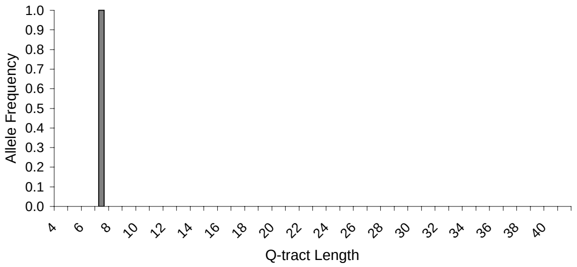

AO. PCQAPa

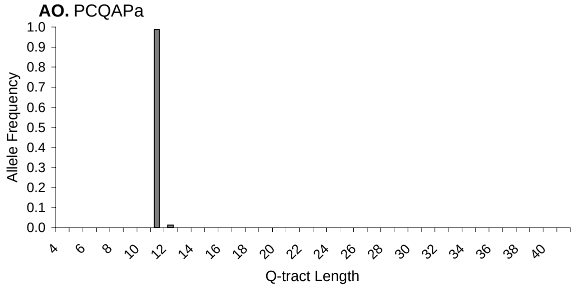

AP. PCQAPb

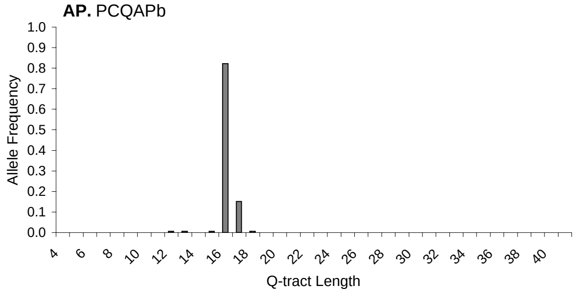

AQ. PHC1

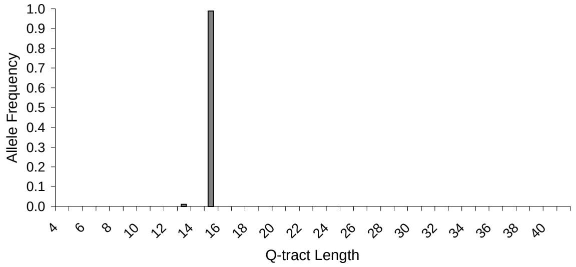

AR. PHLDA1

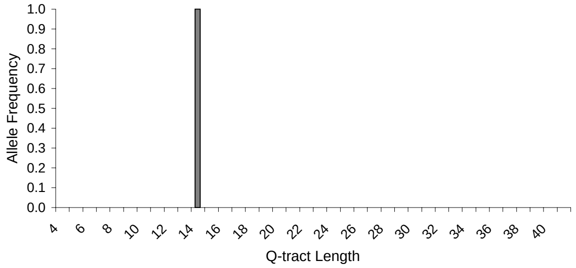

AS. POLG

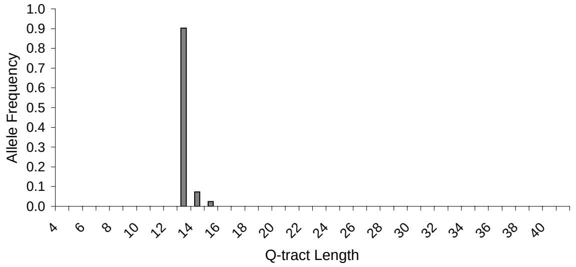

AT. POU3F2

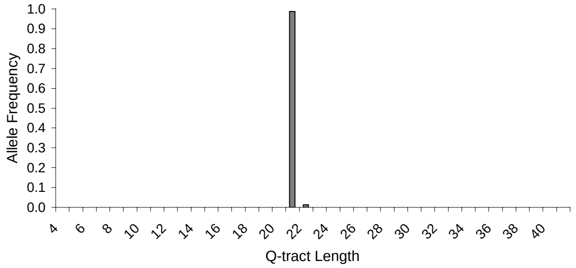

AU. POU6F2

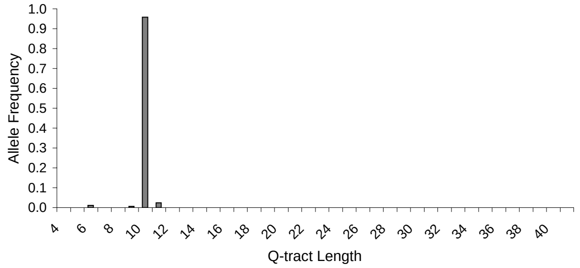

AV. PRDM10

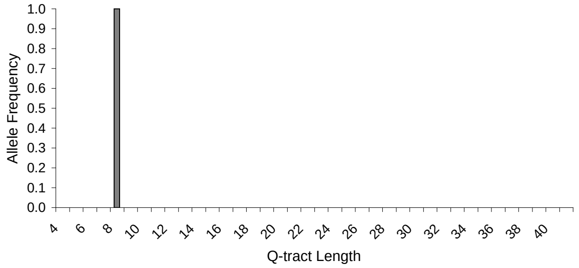

AW. PRKCBP1

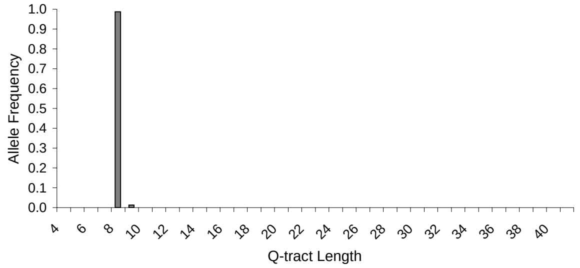

AX. RAI1

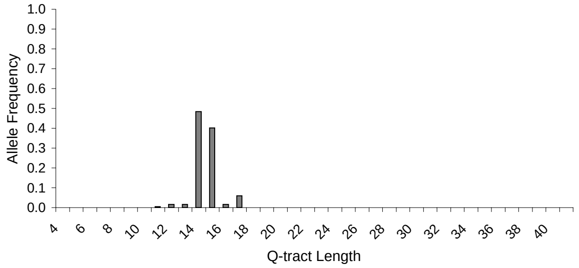

AY. RUNX2

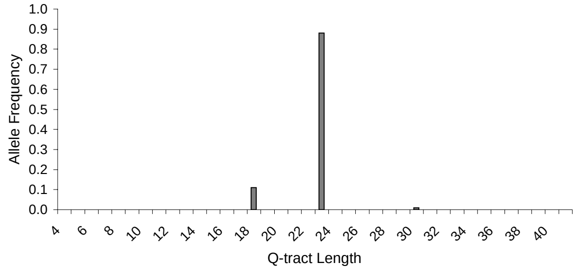

AZ. SATB1

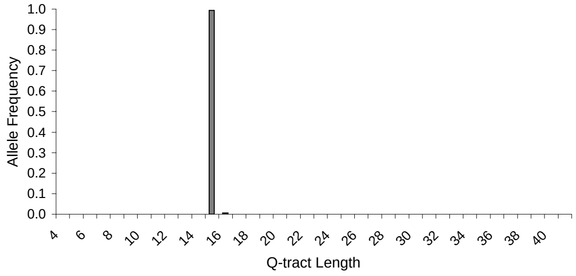

BA. ST6GALNAC5

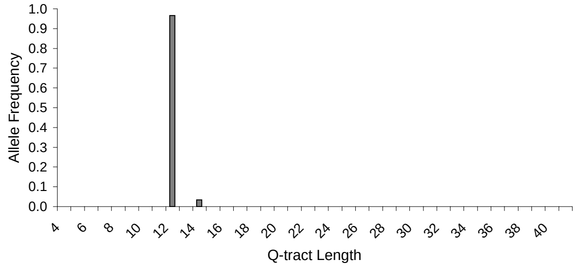

BB. SMARCA2

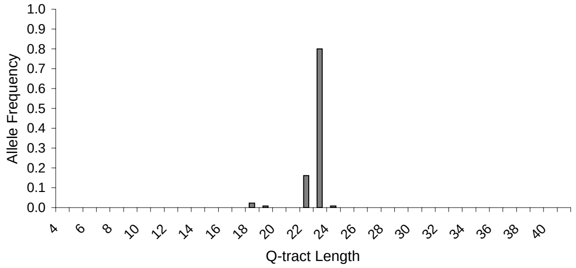

BC. SOCS7

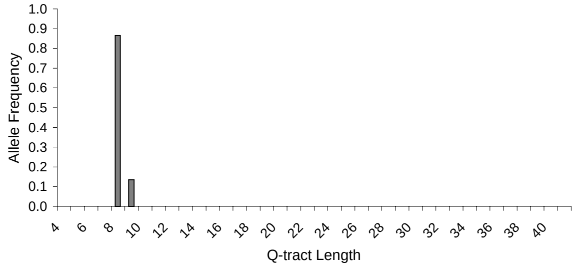

BD. TBP

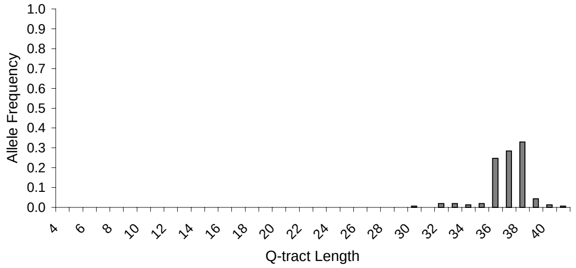

BE. TFEB

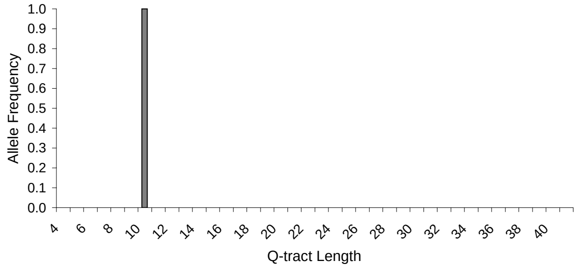

BF. THAP11

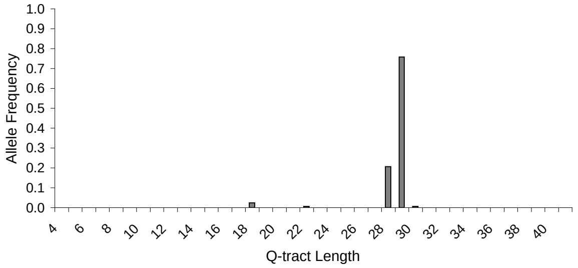

BG. TNRC4

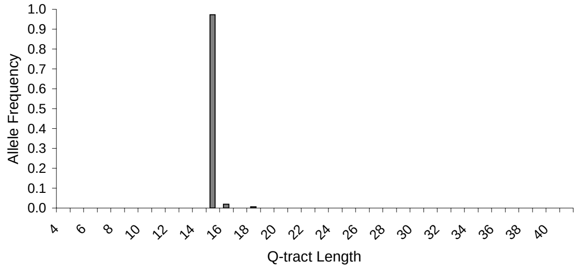

BH. TNRC6A

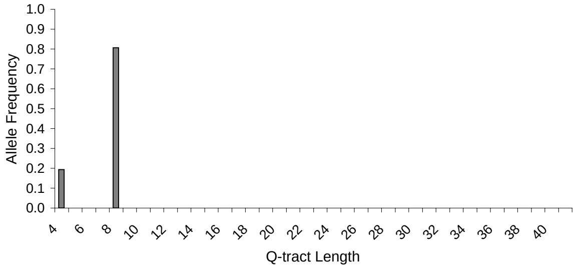

BI. TNRC6B

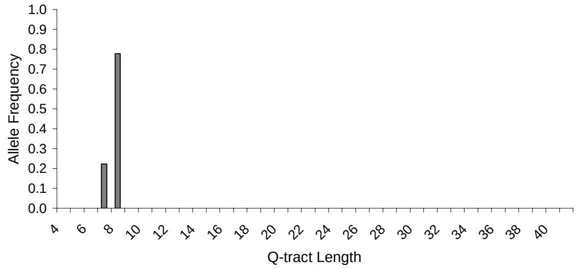

BJ. TNS

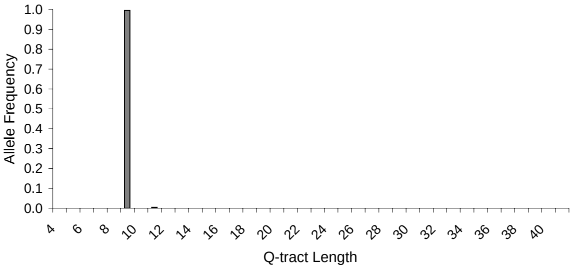

BK. VEZF1

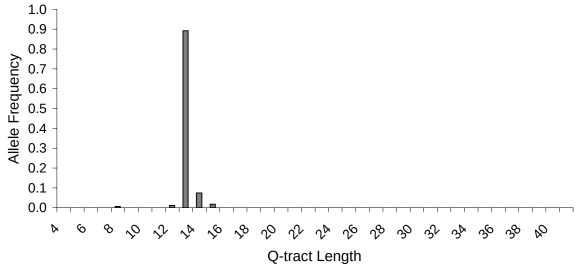

BL. ZNF384

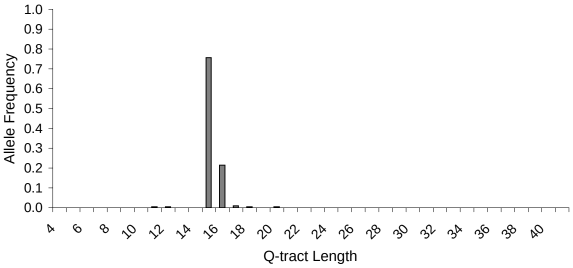

Additional file 2
