# Supplementary material for: CAG-encoded polyglutamine length polymorphism in the human genome
Source: BMC Genomics. 2007 May 22;8:126. doi: 10.1186/1471-2164-8-126 (PMC1896166; doi:10.1186/1471-2164-8-126)
Supplement: Additional file 3 — Genes in over-represented GO terms under Biological Process. For each over-represented GO term and its GO ID, this document lists the CAGpolyQ repeat-containing genes that were annotated with that GO term. [file 1471-2164-8-126-S3.pdf]

Additional file 3. Genes in over-represented GO terms under Biological Process

regulation of biological process GO:0050789
AR|ARID1B|ASCL1|ATXN3|MAGI1|BMP2K|CACNA1A|CHERP|CIZ1|CREBBP|FOXP2|HD|MAML2|MAML3|MED12|MEF2A|MLL2|MN1|NCOA3|NCOA6|NCOR2|NFAT5|PCQAP|PHLDA1|POU3F2|POU6F2|PRDM10|PRKCBP1|RAI1|RUNX2|SATB1|SMARCA2|SOCS7|TBP|TFEB|VEZF1|ZNF384

regulation of physiological process GO:0050791
AR|ARID1B|ASCL1|ATXN3|BMP2K|CACNA1A|CHERP|CIZ1|CREBBP|FOXP2|HD|MAML2|MAML3|MED12|MEF2A|MLL2|MN1|NCOA3|NCOA6|NCOR2|NFAT5|PCQAP|PHLDA1|POU3F2|POU6F2|PRDM10|PRKCBP1|RAI1|RUNX2|SATB1|SMARCA2|SOCS7|TBP|TFEB|VEZF1|ZNF384

regulation of metabolism GO:0019222
AR|ARID1B|ASCL1|ATXN3|CACNA1A|CREBBP|FOXP2|MAML2|MAML3|MED12|MEF2A|MLL2|NCOA3|NCOA6|NCOR2|NFAT5|PCQAP|POU3F2|POU6F2|PRDM10|PRKCBP1|RAI1|RUNX2|SATB1|SMARCA2|TBP|TFEB|VEZF1|ZNF384

positive regulation of metabolism GO:0009893
ARID1B|CREBBP|MAML2|MAML3|NCOA6|NFAT5|RUNX2

nucleobase, nucleoside, nucleotide and nucleic acid metabolism GO:0006139
AR|ARID1B|ASCL1|ATXN3|CACNA1A|CHERP|CIZ1|CREBBP|DCP1B|EP400|FOXP2|MAML2|MAML3|MED12|MEF2A|MLL2|NCOA3|NCOA6|NCOR2|NFAT5|PCQAP|POLG|POU3F2|POU6F2|PRDM10|PRKCBP1|RAI1|RUNX2|SATB1|SMARCA2|TBP|TFEB|VEZF1|ZNF384
