# Supplementary material for: CAG-encoded polyglutamine length polymorphism in the human genome
Source: BMC Genomics. 2007 May 22;8:126. doi: 10.1186/1471-2164-8-126 (PMC1896166; doi:10.1186/1471-2164-8-126)
Supplement: Additional file 4 — Genes in over-represented GO terms under Molecular Function. For each over-represented GO term and its GO ID, this document lists the CAGpolyQ repeat-containing genes that were annotated with that GO term. [file 1471-2164-8-126-S4.pdf]

Additional file 4. Genes in over-represented GO terms under Molecular Function

transcription regulator activity GO:0030528
AR|ARID1B|ASCL1|CREBBP|FOXP2|HD|MAML2|MAML3|MED12|MEF2A|MLL2|NCOA3|NCOA6|NCOR2|NFAT5|POU3F2|POU6F2|RUNX2|SATB1|SMARCA2|TBP|TFEB|VEZF1|ZNF384

transcription cofactor activity GO:0003712
ARID1B|CREBBP|HD|MAML2|MAML3|MED12|MEF2A|NCOA3|NCOA6|NCOR2|SMARCA2

transcription coactivator activity GO:0003713
ARID1B|CREBBP|MAML2|MAML3|MED12|MEF2A|NCOA3|NCOA6|SMARCA2

nucleic acid binding GO:0003676
AR|ARID1B|ARID3B|ASCL1|ATXN1|ATXN7|CACNA1A|CHERP|CIZ1|CREBBP|EP400|FOXP2|MEF2A|MLL2|NCOA6|NCOR2|NFAT5|PHC1|POLG|POU3F2|POU6F2|PRDM10|PRKCBP1|RAI1|RUNX2|SATB1|SMARCA2|TBP|TFEB|THAP11|TNRC4|TNRC6A|TNRC6B|VEZF1|ZNF384

DNA binding GO:0003677
AR|ARID1B|ARID3B|ASCL1|CACNA1A|CREBBP|EP400|FOXP2|MEF2A|MLL2|NCOA6|NCOR2|NFAT5|PHC1|POLG|POU3F2|POU6F2|PRDM10|PRKCBP1|RAI1|RUNX2|SATB1|SMARCA2|TBP|TFEB|THAP11|VEZF1|ZNF384

transcription factor binding GO:0008134
ARID1B|CREBBP|HD|MAML2|MAML3|MED12|MEF2A|NCOA3|NCOA6|NCOR2|SMARCA2|TBP
