# Supplementary material for: CAG-encoded polyglutamine length polymorphism in the human genome
Source: BMC Genomics. 2007 May 22;8:126. doi: 10.1186/1471-2164-8-126 (PMC1896166; doi:10.1186/1471-2164-8-126)
Supplement: Additional file 5 — Genes in over-represented GO terms under Cellular Component. For each over-represented GO term and its GO ID, this document lists the CAGpolyQ repeat-containing genes that were annotated with that GO term. [file 1471-2164-8-126-S5.pdf]

**Additional file 5.** Genes in over-represented GO terms under Cellular Component

organelle GO:0043226
AR|ARID1B|ASCL1|ATXN1|ATXN2|ATXN3|ATXN7|BMP2K|BRD4|CACNA1A|CIZ1|CREBBP|DCP1B|EP400|FOXP2|HD|MAML2|MAML3|MED12|MEF2A|MLL2|NCOA3|NCOA6|NCOR2|NFAT5|PAXIP1|PCQAP|PHC1|PHLDA1|POLG|POU3F2|POU6F2|PRDM10|RAI1|RUNX2|SATB1|SMARCA2|ST6GALNAC5|TBP|TFEB|THAP11|VEZF1|ZNF384

membrane-bound organelle GO:0043227
AR|ARID1B|ASCL1|ATXN1|ATXN2|ATXN3|ATXN7|BMP2K|BRD4|CACNA1A|CIZ1|CREBBP|DCP1B|EP400|FOXP2|HD|MAML2|MAML3|MED12|MEF2A|MLL2|NCOA3|NCOA6|NCOR2|NFAT5|PAXIP1|PCQAP|PHC1|PHLDA1|POLG|POU3F2|POU6F2|PRDM10|RAI1|RUNX2|SATB1|SMARCA2|ST6GALNAC5|TBP|TFEB|THAP11|VEZF1|ZNF384

intracellular GO:0005622
AR|ARID1B|ARID3B|ASCL1|ATXN1|ATXN2|ATXN3|ATXN7|BMP2K|BRD4|CACNA1A|CHERP|CIZ1|CREBBP|DCP1B|EP400|FOXP2|HD|MAGI1|MAML2|MAML3|MED12|MEF2A|MLL2|NCOA3|NCOA6|NCOR2|NFAT5|NUMBL|PAXIP1|PCQAP|PHC1|PHLDA1|POLG|POU3F2|POU6F2|PRDM10|RAI1|RUNX2|SATB1|SMARCA2|ST6GALNAC5|TBP|TFEB|THAP11|VEZF1|ZNF384

intracellular organelle GO:0043229
AR|ARID1B|ASCL1|ATXN1|ATXN2|ATXN3|ATXN7|BMP2K|BRD4|CACNA1A|CIZ1|CREBBP|DCP1B|EP400|FOXP2|HD|MAML2|MAML3|MED12|MEF2A|MLL2|NCOA3|NCOA6|NCOR2|NFAT5|PAXIP1|PCQAP|PHC1|PHLDA1|POLG|POU3F2|POU6F2|PRDM10|RAI1|RUNX2|SATB1|SMARCA2|ST6GALNAC5|TBP|TFEB|THAP11|VEZF1|ZNF384

intracellular membrane-bound organelle GO:0043231
AR|ARID1B|ASCL1|ATXN1|ATXN2|ATXN3|ATXN7|BMP2K|BRD4|CACNA1A|CIZ1|CREBBP|DCP1B|EP400|FOXP2|HD|MAML2|MAML3|MED12|MEF2A|MLL2|NCOA3|NCOA6|NCOR2|NFAT5|PAXIP1|PCQAP|PHC1|PHLDA1|POLG|POU3F2|POU6F2|PRDM10|RAI1|RUNX2|SATB1|SMARCA2|ST6GALNAC5|TBP|TFEB|THAP11|VEZF1|ZNF384

nucleus GO:0005634
AR|ARID1B|ASCL1|ATXN1|ATXN2|ATXN3|ATXN7|BMP2K|BRD4|CACNA1A|CIZ1|CREBBP|DCP1B|EP400|FOXP2|HD|MAML2|MAML3|MED12|MEF2A|MLL2|NCOA3|NCOA6|NCOR2|NFAT5|PAXIP1|PCQAP|PHC1|PHLDA1|POU3F2|POU6F2|PRDM10|RAI1|RUNX2|SATB1|SMARCA2|TBP|TFEB|THAP11|VEZF1|ZNF384

nucleoplasm GO:0005654
ARID1B|ATXN3|CREBBP|MED12|NCOA6|NFAT5|PHC1|POU3F2|SMARCA2|TBP|TFEB
