# Supplementary material for: CAG-encoded polyglutamine length polymorphism in the human genome
Source: BMC Genomics. 2007 May 22;8:126. doi: 10.1186/1471-2164-8-126 (PMC1896166; doi:10.1186/1471-2164-8-126)
Supplement: Additional file 6 — Genes and their shared GO terms under Cellular Component. This document provides GO IDs, their descriptions, and the lists of CAGpolyQ repeat-containing genes that shared these annotations above the 99th percentile cutoff. [file 1471-2164-8-126-S6.pdf]

Additional file 6. Genes and their shared GO terms under Cellular Component

GO:0005654 nucleoplasm
ATXN3|NCOA6|SMARCA2

GO:0005667 transcription factor complex
NCOA6|TBP

GO:0008372 cellular component unknown
CXORF6|MN1|SOCS7
