# Supplementary material for: CAG-encoded polyglutamine length polymorphism in the human genome
Source: BMC Genomics. 2007 May 22;8:126. doi: 10.1186/1471-2164-8-126 (PMC1896166; doi:10.1186/1471-2164-8-126)
Supplement: Additional file 7 — Genes and their shared GO terms under Biological Process. This document provides GO IDs, their descriptions, and the lists of CAGpolyQ repeat-containing genes that shared these annotations above the 99th percentile cutoff. [file 1471-2164-8-126-S7.pdf]

Additional file 7. Genes and their shared GO terms under Biological Process

GO:0000004 biological process unknown
ARID3B|C14ORF4|CXORF6

GO:0000074 regulation of cell cycle
CIZ1|MN1

GO:0000398 nuclear mRNA splicing, via spliceosome
CIZ1|TNRC4

GO:0006260 DNA replication
NCOA6|POLG

GO:0006281 DNA repair
ATXN3|NCOA6

GO:0006325 establishment and/or maintenance of chromatin architecture
EP400|SATB1

GO:0006350 transcription
AR|ASCL1|ATXN3|FOXP2|MED12|MEF2A|MLL2|NCOA6|PCQAP|PRDM10|RUNX2|
SMARCA2|TBP|VEZF1|ZNF384

GO:0006355 regulation of transcription, DNA-dependent
AR|ASCL1|ATXN3|CREBBP|FOXP2|MED12|MEF2A|MLL2|NCOA3|NCOA6|NCOR2|NFAT5|
PCQAP|POU3F2|POU6F2|PRDM10|PRKCBP1|RAI1|RUNX2|SATB1|SMARCA2|TBP|TFEB|
VEZF1|ZNF384

GO:0006357 regulation of transcription from RNA polymerase II promoter
ASCL1|NCOA6|SMARCA2|VEZF1

GO:0006366 transcription from RNA polymerase II promoter
ASCL1|MED12|MEF2A|MLL2|NCOA6|NFAT5|POU6F2|SMARCA2|TBP|VEZF1

GO:0006367 transcription initiation from RNA polymerase II promoter
MED12|NCOA6|TBP

GO:0006461 protein complex assembly
MAGI1|CREBBP

GO:0006468 protein amino acid phosphorylation
BMP2K|MINK1

GO:0006486 protein amino acid glycosylation
KIAA2018|ST6GALNAC5

GO:0007268 synaptic transmission
ATXN3|CACNA1A|KCNN3

GO:0007399 neurogenesis
ASCL1|ATN1|ATXN3|CACNA1A|CHERP|KCNN3|MAML3|NUMBL|POU6F2|TNRC4|ZNF384

GO:0007417 central nervous system development
ATN1|NCOA6|POU6F2

GO:0007601 visual perception
ATXN7|POU6F2

GO:0045449 regulation of transcription
AR|ASCL1|ATXN3|CREBBP|FOXP2|MED12|MEF2A|MLL2|NCOA3|NCOA6|NCOR2|NFAT5|
PCQAP|POU3F2|POU6F2|PRDM10|PRKCBP1|RAI1|RUNX2|SATB1|TBP|TFEB|ZNF384
