# Supplementary material for: CAG-encoded polyglutamine length polymorphism in the human genome
Source: BMC Genomics. 2007 May 22;8:126. doi: 10.1186/1471-2164-8-126 (PMC1896166; doi:10.1186/1471-2164-8-126)
Supplement: Additional file 8 — Genes and their shared GO terms under Molecular Function. This document provides GO IDs, their descriptions, and the lists of CAGpolyQ repeat-containing genes that shared these annotations above the 99th percentile cutoff. [file 1471-2164-8-126-S8.pdf]

Additional file 8. Genes and their shared GO terms under Molecular Function

GO:0003677 DNA binding
AR|ARID1B|ARID3B|ASCL1|CREBBP|EP400|FOXP2|MAML3|MED12|MEF2A|MLL2|NCOR2|NFAT5|PHC1|POLG|POU3F2|POU6F2|PRDM10|PRKCBP1|RAI1|RUNX2|SATB1|SMARCA2|TBP|TFEB|THAP11|TNRC4|VEZF1|ZNF384

GO:0003700 transcription factor activity
AR|ASCL1|CREBBP|FOXP2|MAML3|MEF2A|MLL2|NFAT5|POU3F2|POU6F2|RUNX2|SATB1|TFEB|TNRC4

GO:0003713 transcription coactivator activity
ARID1B|CREBBP|MED12|MEF2A|NCOA3|NCOA6|SMARCA2

GO:0003714 transcription corepressor activity
HD|NCOR2

GO:0004402 histone acetyltransferase activity
CREBBP|NCOA3

GO:0004674 protein serine/threonine kinase activity
BMP2K|MINK1

GO:0005509 calcium ion binding
CACNA1A|KIAA2018

GO:0005524 ATP binding
MAGI1|BMP2K|EP400|MED12|MINK1|RUNX2|SMARCA2

GO:0005554 molecular function unknown
ATXN2|CXORF6|MN1

GO:0008270 zinc ion binding
ATXN7|CIZ1|CREBBP|FOXP2|MLL2|PRDM10|VEZF1|ZNF384

GO:0016251 general RNA polymerase II transcription factor activity
MED12|TBP

GO:0016563 transcriptional activator activity
MED12|NCOA6

GO:0030374 ligand-dependent nuclear receptor transcription coactivator activity
MED12|NCOA6

GO:0046966 thyroid hormone receptor binding
MED12|NCOA3|NCOA6
